# Fine‐Tuning Porous Structure of Zirconium‐Based Metal–Organic Frameworks for Efficient Separation and Purification of Astaxanthin by Defect Engineering

**DOI:** 10.1002/advs.202409451

**Published:** 2024-11-14

**Authors:** Xin Na, Shanghua Xing, Mingqian Tan, Wentao Su

**Affiliations:** ^1^ State Key Laboratory of Marine Food Processing and Safety Control Dalian Polytechnic University Dalian Liaoning 116034 China; ^2^ Academy of Food Interdisciplinary Science School of Food Science and Technology Dalian Polytechnic University Dalian Liaoning 116034 China; ^3^ National Engineering Research Center of Seafood Dalian Polytechnic University Dalian Liaoning 116034 China; ^4^ Collaborative Innovation Center of Seafood Deep Processing Dalian Polytechnic University Dalian 116034 China

**Keywords:** astaxanthin, metal–organic frameworks, purification, separation

## Abstract

Efficient separation of bioactive compounds from nature source, particularly that of astaxanthin (AXT), remains challenging due to their low content in complicated matrix and readily degradable structure. Herein, a modulator‐induced defect engineering is presented on the stable zirconium‐based metal–organic frameworks (Zr‐MOFs) to optimize pore size and pore chemistry for the efficient separation and purification of AXT for the first time. High adsorption capacity of 26.21 mg g^−1^ is achieved on the best‐performing defect Zr‐MOF (d‐UiO‐67‐4), superior over the other reported adsorbent for AXT. Meanwhile, d‐UiO‐67‐4 exhibits the selective adsorption of AXT over other carotenoids analogues with similar structure and properties. This is attributed to the preferential non‐covalent interactions between defect framework and AXT revealed by the spectroscopy analysis and density functional theory (DFT) calculations. High purity of AXT with 89.0% ± 2.3% extraction efficiency can be realized after the purification of AXT by d‐UiO‐67‐4. The practical separation performance of d‐UiO‐67‐4 for AXT extracted from *Haematococcus pluvialis* is demonstrated by fixed‐bed column‐based dynamic adsorption and desorption experiments. This work broadens the preparation methods for thermosensitive active substances and provided new research ideas for the controlled adsorption of functional food factors.

## Graphical abstract

This study reports for the first time to achieve the optimal pore environment of MOFs with the multiple synergetic host‐guest interactions for the efficient separation and purification of astaxanthin (AXT) from *Haematococcus pluvialis* by controlling modulator‐induced defect. This work indicates the great potential of MOFs for production, including extraction and separation, of astaxanthin from nature source.

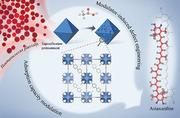

## 1 Introduction

Astaxanthin (AXT) is a keto‐carotenoid belong to the xanthophylls class abundant in various water‐living organisms.^[^ ^1^ ^]^ AXT possess the excellent antioxidant properties with ≈10‐ and 100‐fold enhancement over other carotenoids and α‐tocopherol.^[^ ^2^ ^]^ Besides, AXT has the anti‐inflammatory, anti‐tumor, and cardiovascular healthy effect.^[^ ^3^ ^]^ Nature AXT has a high market value with 2500–7000 $ kg^−1^ and has been marketed as the dietary supplement approved by the United States Food and Drug Administration.^[^ ^4^ ^]^ Due to their high market value and superior biological activities, AXT has a great demand in food and pharmaceutical applications, and many efforts have been devoted into the recovery and separation of AXT from nature sources.^[^ ^5^ ^]^

Conventional extraction of AXT from nature sources mainly include solvent extraction, followed by separation and purification.^[^ ^6^ ^]^ Adsorption of target compound from crude extracts has been proven as a promising efficient separation method for the purification of bioactive compounds due to the easy operation, economic feasibility, and negligible secondary pollution.^[^ ^7^ ^]^ Traditional materials of silica gels and microporous resins have been widely developed as the adsorbent for bioactive compounds.^[^ ^8^ ^]^ AXT has the hydrophobic hydrocarbons C40 skeleton and polar hydroxyl and keto groups in terminal rings.^[^ ^9^ ^]^ The inherent limitations of traditional materials in terms of low porosity and inferior adsorption interactions generally result in the low selectivity and adsorption capacity.^[^ ^8^ ^]^ Therefore, an ideal adsorbent for AXT separation should possess the larger pore diameter over the molecular size of AXT (33.97 × 9.02 Å) and the preferential binding sites involving hydrophobic or hydrogen bonding interactions with AXT.^[^ ^10^ ^]^

In this aspect, metal–organic frameworks (MOFs) offer great promise for separation and purification due to their high tunability in their pore size and surface functionality.^[^ ^11^ ^]^ Moreover, defect engineering has been recently recognized as a useful strategy for tuning the structure properties of MOFs.^[^ ^12^ ^]^ The defect in MOFs can be achieved by incorporating the acid modulators in synthesis, preferentially binding with metal center to replace the linker, leading to the missing linker defects in structure with the enlarged pore size and altered pore chemistry.^[^ ^13^ ^]^ The defects can be engineered to optimize the MOFs pore size ranging from micro‐ (< 2 nm), meso‐ (2–50 nm) to macroporosity (> 50 nm).^[^ ^14^ ^]^ At the same time, the pore surface chemistry with different polarities can be tailored to enhance the host‐guest interactions.^[^ ^15^ ^]^ Defect‐engineered MOFs have shown the superior separation efficiencies for a variety of guest compounds ranging from small gas molecules, e.g., C_2_H_2_, C_3_H_6_ to large molecules, e.g., polycyclic aromatic hydrocarbons, perfluorinated alkyl substances.^[^ ^16^ ^]^ However, the study on the recovery and separation of AXT by defective MOFs was still unexplored.

Zirconium‐based MOFs (Zr‐MOFs) are an important class of MOFs due to their superior chemical/thermal stabilities and nontoxicity compared to other transitional metal‐based MOFs.^[^ ^17^ ^]^ An ideal UiO‐67 as a representative Zr‐MOF is constituted of Zr_6_O_4_(*μ*_3_‐OH)_4_^12+^ clusters connected by biphenyl‐dicarboxylate (BPDC) linkers.^[^ ^18^ ^]^ Notably, UiO‐67 is high tolerance for defect structure modulation due to its inherent high structure stability.^[^ ^19^ ^]^ An ideal UiO‐67 has octahedral and tetrahedral cavities with the pore diameter of 17.5 and 11.5 Å, respectively. And its two cavities are linked by the triangular pore aperture of 8 Å.^[^ ^20^ ^]^ Considering its hydrophobic pore structure, available adsorption sites (e.g., ─OH groups), and the large pore cavity, we propose that UiO‐67 could be a good candidate for AXT adsorption through hydrophobic interaction and hydrogen bonding. On the other hand, the pore size and pore surface chemistry with different hydrophobicity can be tuned for enhanced AXT separation by modulator‐induced defect engineering.^[^ ^21^ ^]^

In this work, a series of UiO‐67 with different content of defect (d‐UiO‐67‐X, X = 2, 4, 6, and 8, which represent the molar ratio of acetic acid to BPDC in synthesis) were prepared by modulator‐induced defect engineering strategy. The acetic acid is employed as the modulator and its concentration in synthesis is closely related to the formation of defect content in d‐UiO‐67‐X. The modulation of structure defects in d‐UiO‐67‐X realize the fine‐tuning of pore size, internal surface area, and adsorption sites, which highly influence on the adsorption and desorption properties of AXT. The insight on AXT adsorption mechanism was investigated by Density‐Functional Theory (DFT) calculations and Fourier Transform Infrared Spectroscopy (FT‐IR) spectra. Finally, we explored the practical performance of d‐UiO‐67‐X for the separation and purification of target AXT from the crude extract of *Haematococcus pluvialis* powder.

## 2 Results and Discussion

### 2.1 Characterization of d‐UiO‐67‐X Series

**Figure 1:**
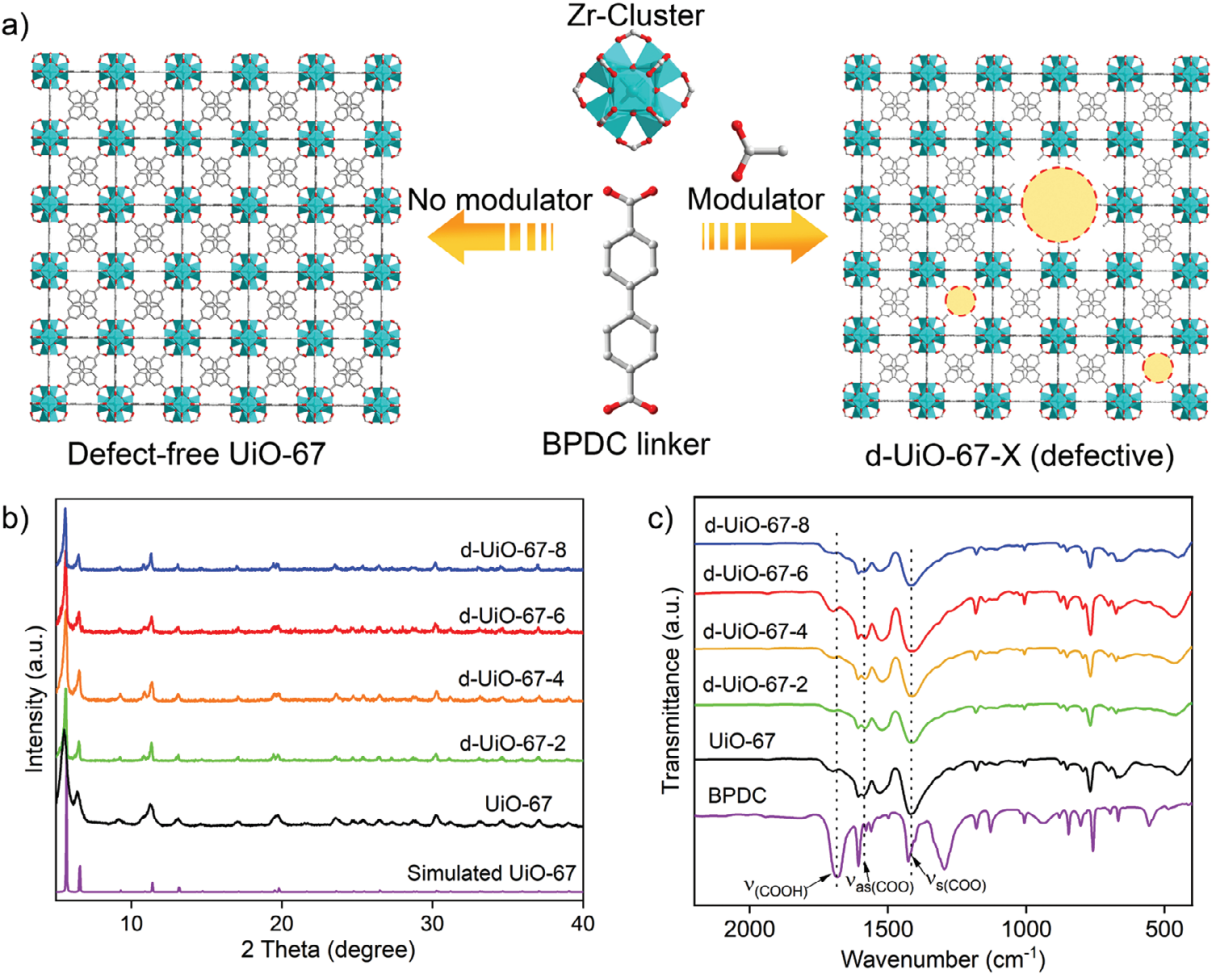
a) Schematic illustration of defective‐free UiO‐67 and d‐UiO‐67‐X; PXRD patterns b) and FT‐IR spectra c) of UiO‐67 and d‐UiO‐67‐X.

The acetate acid as modulator was incorporated into the structure of UiO‐67 to partially substitute the BPDC linker for the formation of defect d‐UiO‐67‐X (**Figure** **1**a), X represent the molar ratio of acetate acid/BPDC with 2, 4, 6, and 8. As shown in Figure 1b, the d‐UiO‐67‐X series showed the similar PXRD patterns with the pristine UiO‐67 and consistent with the simulated one that obtained from the single crystal structure data (CCDC No. 2179856).^[^ ^22^ ^]^ This indicates that topology structure was retained without the phase transformation after introducing the modulator of acetic acid in synthesis. Compared to parent UiO‐67, the diffraction peak of (111) and (200) plane for the PXRD patterns of d‐UiO‐67‐X slightly shifted to the lower angle, which indicated the increased interplanar spacing of d‐UiO‐67‐X. And the peak of d‐UiO‐67‐X turned the wider with the decreased crystallinity. From the SEM images shown in Figures S1–S5 (Supporting Information), the apparent particle agglomeration phenomenon was found in UiO‐67, and the morphologies of d‐UiO‐67‐X showed clear octahedral crystal particles. The EDS elemental mapping (Figures S1–S5, Supporting Information) shows the uniform dispersion of Zr, C, and O species in UiO‐67 and d‐UiO‐67‐X. As revealed by FT‐IR spectra (Figure 1c), compared to UiO‐67, the asymmetric and symmetric stretching vibration peaks of COO^−^ for d‐UiO‐67‐X were red‐shifted. This is probably due to the partial substituent of BPDC ligand by mono‐carboxylate modulator of acetate in structure.

**Figure 2:**
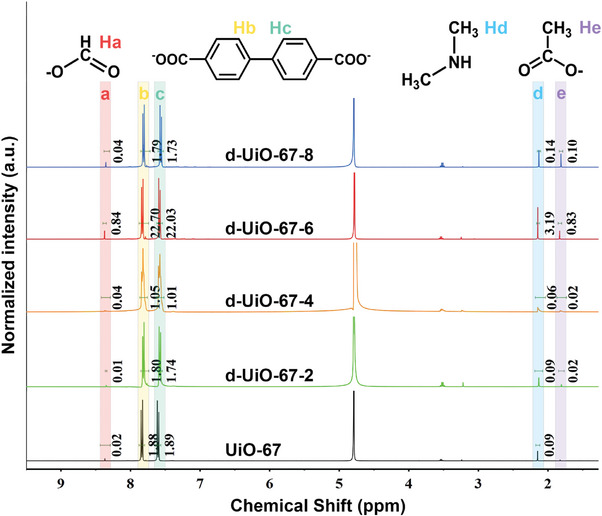
^1^H NMR spectra of UiO‐67 and d‐UiO‐67‐X.

The number of defects in framework was determined from ^1^H NMR spectra of digested d‐UiO‐67‐X samples (**Figure** **2**; Figure S6, Supporting Information). All the d‐UiO‐67‐X have two significant signals in the ^1^H NMR spectra, which are assigned to BPDC (*δ* = 7.85 and 7.60 ppm, 8H) and acetate (*δ* = 1.84 ppm, 3H). The acetate signal was absent in digested UiO‐67. The signals of formate (*δ* = 8.38 ppm, H) and dimethylamine (*δ* = 2.13 ppm, 6H) in UiO‐67 and d‐UiO‐67‐X originate from the hydrolysis of DMF solvent in synthesis, which was also found in other reported digested MOFs.^[^ ^23^ ^]^ As increasing the amount of acetic acid in synthesis, the molar ratio of acetate/BPDC accordingly increase by integrating area of ^1^H NMR peaks in d‐UiO‐67‐X, indicating that the more defect sites are formed by incorporating acetate modulators in structure.

**Figure 3:**
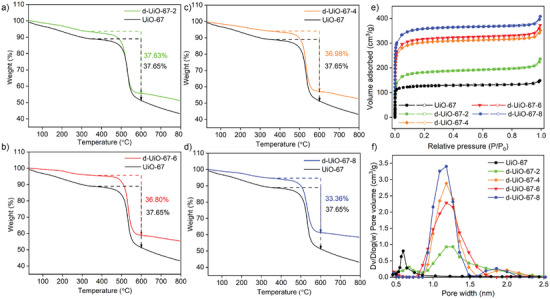
TGA curves of UiO‐67 and d‐UiO‐67‐X a–d); N_2_ sorption isotherms at 77 K e) and Pore size distribution f) of d‐UiO‐67‐X.

The results of TGA curves of d‐UiO‐67‐X can further demonstrate the structural defect induced by incorporating the modulator of acetic acid. As shown in **Figure** **3**a–d, the TGA curves of UiO‐67‐X series and UiO‐67 have three main weight loss stages. The first weight loss at 35–250 °C involve the removal of solvent molecules. The second weight loss at 250–400 °C correspond to the dihydroxylation and the loss of acetate modulator linkers from Zr_6_ clusters. The third weight loss starting from 500 °C was due to the framework collapse followed by BPDC linker decomposition. The smaller weight loss (%) of BPDC linker in UiO‐67‐X was found compared to UiO‐67 (37.65%). The weight loss (%) of BPDC linker at 400–600 °C for d‐UiO‐67‐2, d‐UiO‐67‐4, d‐UiO‐67‐6, and d‐UiO‐67‐8 was 37.63%, 36.98%, 36.80%, and 33.36%, respectively. This reveals the increased missing BPDC linkers by increasing the amount of acetate modulator linkers in structure.

The effect of acetate modulator linker on the porosity structure was investigated by nitrogen sorption at 77 K. As shown in Figure 3e, the N_2_ adsorption–desorption isotherms of UiO‐67 and UiO‐67‐X showed the type‐I isotherms indicating the typical microporous materials. With the increase of acetic acid, the BET surface area of UiO‐67‐X was increased. As shown in Table S1 (Supporting Information), the BET surface area of UiO‐67 was determined to be 503.8 m^2^ g^−1^, while that of UiO‐67‐2, UiO‐67‐4, UiO‐67‐6, and UiO‐67‐8 was 674.5, 1153.0, 1206.3, and 1364.1 m^2^ g^−1^, respectively. The increased BET surface area of UiO‐67‐X by increasing acetate modulators indicates the formation of the more defect in structure, compared to UiO‐67 (503.8 m^2^ g^−1^). The pore size distribution (Figure 3f) of UiO‐67 showed the maximum pore width at 0.6 nm, while d‐UiO‐67‐X had the obviously increased pore width at ≈1.25 nm with the expanded pore size in the range of 1.7–2.1 nm.

### 2.2 Adsorption Properties of d‐UiO‐67‐X Series for AXT

The adsorption properties of d‐UiO‐67‐X for AXT were explored by recording the characteristic adsorption peak of AXT at 480 nm by UV–vis spectroscopy, as shown in Figures S7–S11 (Supporting Information). The gradual color fading of AXT solution and the color change of d‐UiO‐67‐X powder from white to orange revealed the AXT adsorbed by d‐UiO‐67‐X. The structure of AXT is known to be sensitive with the possible skeleton degradation under external environment conditions.^[^ ^24^ ^]^ In order to further prove the AXT adsorbed by d‐UiO‐67‐X, instead of color fading of AXT solution induced by structure degradation of AXT, UV–vis spectra of pure AXT solution under the same adsorption condition by avoiding light irradiation for 24 h was measured and compared with that of initial AXT solution. The nearly unchanged AXT characteristic adsorption peak intensity from UV–vis spectra (Figure S12, Supporting Information) supports the successful AXT adsorption onto d‐UiO‐67‐X. The standard curve of AXT solution with the concentration of 2–8 mg L^−1^ was built (Figure S13, Supporting Information). In addition, PXRD patterns (Figure S14, Supporting Information) and SEM images of AXT incorporated d‐UiO‐67‐X were not obviously changed compared with their pristine d‐UiO‐67‐X (Figures S15–S18, Supporting Information), reflecting the framework stability after AXT adsorption.

**Figure 4:**
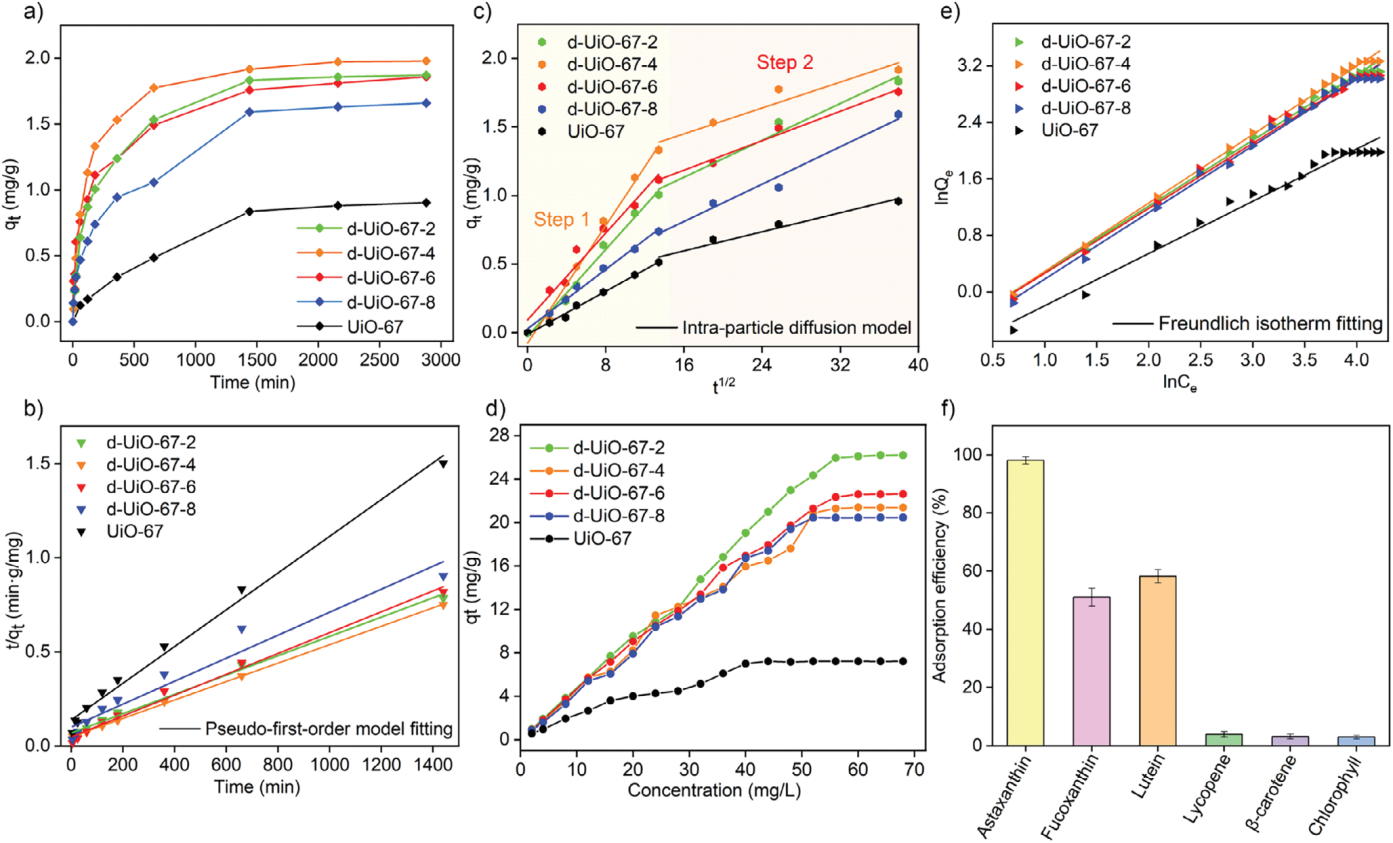
a) The time‐dependent adsorption capacity curves, b) Pseudo‐first‐order model fitting, c) intra‐particle diffusion model fitting, d) AXT concentration dependent adsorption capacity curves, and e) Freundlich isotherm fitting of UiO‐67 and d‐UiO‐67‐X; f) Adsorption selectivity of d‐UiO‐67‐4.

The effect of time on adsorption capacity was investigated when the AXT concentration was 4 mg L^−1^. As shown in **Figure** **4**a, for all d‐UiO‐67‐X, the adsorption rate was fast during the beginning 500 min, and then achieve the adsorption equilibrium as the adsorption time was up to ≈1500 min. Obviously, compared to UiO‐67, the faster adsorption rate and the higher adsorption equilibrium capacity was found in all d‐UiO‐67‐X. This can be attributed to the higher BET surface area and the more active adsorption sites in d‐UiO‐67‐X due to the formation of defect structure induced by the incorporation of acetate modulators. The adsorption uptake of UiO‐67 for AXT was 0.96 mg g^−1^, which was significantly lower than that of d‐UiO‐67‐X. In addition, as increasing the molar ratio of acetic acid/BPDC from 2 to 4, the adsorption uptake of AXT increase from 1.83 mg g^−1^ in d‐UiO‐67‐2 to 1.92 mg g^−1^ in d‐UiO‐67‐4. However, if the ratio of acetic acid/BPDC further increase to 6 and 8, the AXT adsorption uptakes on d‐UiO‐67‐6 and d‐UiO‐67‐8 decrease to 1.76 and 1.60 mg g^−1^, respectively. As such, d‐UiO‐67‐4 behave the best adsorption performance by comparing the adsorption rate and adsorption capacity with other three d‐UiO‐67‐X materials. The above results indicate the importance of tuning the defect structure of d‐UiO‐67‐X by different amount of acetate modulators to maximally enhance the adsorption performance of AXT.

The adsorption kinetics of time‐dependence adsorption process was determined by employing different kinetic models including the pseudo‐first order, pseudo‐second order, and intraparticle diffusion models. The fitted kinetic parameters are shown in Tables S2–S11 (Supporting Information). And the fitting curves are shown in Figure 4b and Figures S19–S24 (Supporting Information). Compared to pseudo‐first order models, the higher coefficient R^2^ was present in pseudo‐second order model fitted data for d‐UiO‐67‐X with different AXT concentrations (2, 4, and 8 mg L^−1^). This indicates adsorption process of d‐UiO‐67‐X for AXT was mainly controlled by chemical adsorption. The intraparticle diffusion models were further used to describe diffusion‐controlled adsorption kinetics. By plotting the *q_t_* versus *t* ^0.5^, multilinearity with two slopes indicates that AXT adsorption onto d‐UiO‐67‐X and UiO‐67 involve two‐stage adsorption process (Figure 4c). The diffusion constants in the first and second stage for d‐UiO‐67‐X were significantly higher than UiO‐67, indicating that the enlarged pore size and higher BET surface area induced by defect structure promote the high diffusion of AXT into the pores of d‐UiO‐67‐X. In addition, by comparison of the two‐stage diffusion constants, the diffusion constant in the first stage was obviously higher than that in the second stage. It can be explained by the following: at the beginning adsorption, there are sufficient adsorption active sites that can interact with AXT. Meanwhile, large accessible pore volume is available for AXT diffusion. When most of the adsorption sites and pore volume are occupied by AXT, the adsorption diffusion rate was thus inevitably decreased.

The effect of different AXT concentration on adsorption capacity was explored on d‐UiO‐67‐X and UiO‐67. The adsorption isotherms were shown in Figure 4d. As increasing AXT concentration, adsorption capacity was accordingly increased on d‐UiO‐67‐X and UiO‐67. When AXT concentration was 56 mg L^−1^, adsorption saturation was achieved. The maximum adsorption capacity with 26.21 mg g^−1^ was found in d‐UiO‐67‐4, which was obviously higher than UiO‐67 (7.22 mg g^−1^), other three d‐UiO‐67‐X (20.46–22.62 mg g^−1^) and other reported AXT resin materials as shown in Table S14 (Supporting Information), such as XDA‐8 (2.4 mg g^−1^), LX‐68G (2.7 mg g^−1^), and D101 (2.5 mg g^−1^).^[^ ^25^ ^]^ As shown in Figure 4e and Figure S25 (Supporting Information), by fitting the Freundlich and Langmuir isotherms models with adsorption isotherms of d‐UiO‐67‐X, Freundlich model fitted data (Table S12, Supporting Information) had the higher coefficient *R* ^2^, which indicate the multi‐layer adsorption on the heterogeneous surfaces.

### 2.3 Selective Adsorption of d‐UiO‐67‐X Series for AXT

AXT is generally found in microbial matrix, containing the other nature pigment such as chlorophyll, lutein, and β‐carotene that can influence on the selective adsorption properties of adsorbent for AXT from the mixed matrix.^[^ ^26^ ^]^ From the above results, d‐UiO‐67‐4 with the better adsorption performance was selected for the subsequent selective adsorption measurement. The adsorption capability of d‐UiO‐67‐4 for other carotenoid with the similar structure including fucoxanthin, lutein, lycopene and β‐carotene as well as the commonly existed chlorophyll in microorganism was explored. As shown in Figure 4f, the adsorption efficiencies of fucoxanthin (51.06%) and lutein (58.29%) were higher than that of lycopene (3.92%), β‐carotene (3.17%) and chlorophyll (2.96%), but still significantly lower than that of AXT (98.14%). It should be noted that AXT, fucoxanthin and lutein has the basic structure of tetraterpene with the terminal polar groups containing oxygen atoms. While for lycopene and β‐carotene, they have no oxygen atoms in their tetraterpene structure. This indicates the hydrophobic interaction is not the main adsorption driving force. The other non‐covalent interaction such as hydrogen bonding between d‐UiO‐67‐4 and AXT probably play a key role in the high adsorption performance of AXT.

### 2.4 Adsorption Mechanism of AXT

To unravel the possible interactions of adsorbed AXT with the d‐UiO‐67‐4, FT‐IR spectra and XPS spectra of AXT incorporated d‐UiO‐67‐4 was explored and compared with the pristine d‐UiO‐67‐4. From the FT‐IR spectra shown in Figure S26 (Supporting Information), several vibration modes on the framework of d‐UiO‐67‐4 were changed after AXT adsorption. The asymmetric and symmetric stretching vibrations of COO^−^ on d‐UiO‐67‐4 were blue‐shifted from 1519 and 1408 cm^−1^ to 1525 and 1418 cm^−1^. In addition, the stretching vibration of ─OH at 3419 cm^−1^ were blue‐shifted to 3426 cm^−1^. This reveals the presence of intermolecular interactions between COO^−^ and ─OH on d‐UiO‐67‐4 and AXT. The COO^−^ and ─OH vibration change was also observed on the other three d‐UiO‐67‐X after AXT adsorption, but slight blue‐shift on these peaks were observed, which indicates their interactions with AXT is relatively weak compared to d‐UiO‐67‐4. From the O1s XPS spectra of d‐UiO‐67‐4 shown in Figure S27 (Supporting Information), the peaks at 530.2, 531.7 and 533.3 eV correspond to Zr‐O‐Zr, Zr‐O─C and COOH, respectively. After AXT adsorption, the peaks of Zr─O─Zr and Zr─O─C in the secondary building units of d‐UiO‐67‐4 shifted to 530.5 and 531.9 eV. Within an instrument error of 2 eV, we caution to overinterpret such values, but such change was consistent with the results of FT‐IR spectra.

**Figure 5:**
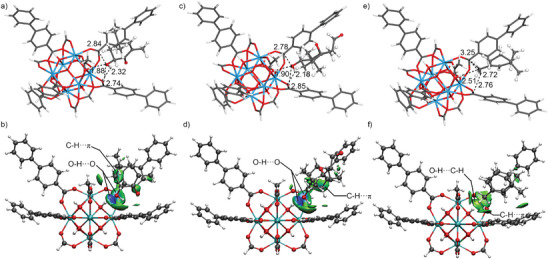
DFT calculated binding sites and IGMH isosurface maps of AXT a,b), fucoxanthin c,d) and β‐carotene e,f) on optimized defective UiO‐67 cluster model.

The favorable interactions of defective UiO‐67 with AXT over other carotenoids were illustrated by dispersion corrected DFT calculations using Gaussian 16 software. Fucoxanthin and β‐carotene belong to the class of carotenoids that are chosen as the representative xanthophyll (contain oxygen) and carotene (contain no oxygen). The main structure difference in carotenoid molecules is the terminal rings. Thus, different terminal ring models were isolated from the optimized DFT structure of AXT, fucoxanthin and β‐carotene (Figure S28, Supporting Information). To mimic the defect pore environment, the isolated cluster models from crystal structure of UiO‐67 were DFT optimized by partial substitution of BPDC linker with acetate modulator (Figure S29, Supporting Information). As shown in **Figure** **5**a, AXT is stabilized in defective UiO‐67 by the enhanced O─H(^δ+^)···(^δ−^)O and O─C(^δ+^)···(^δ−^)O electrostatic attractions. The optimized H···O and C···O distances of 1.88 and 2.74 Å between hydroxyl groups of AXT and μ_3_‐OH of metal cluster are remarkably shorter than the sum of vdW radii of H and O atoms (2.32 Å) as well as that of C and O atoms (3.63 Å). Furthermore, their optimized H···O (1.88 Å) and C···O distances (2.74 Å) are obviously shorter than those (1.90 and 2.85 Å) with fucoxanthin (Figure 5c). For the optimized structure of β‐carotene with defective UiO‐67 (Figure 5e), there are multiple weak O─H(^δ+^)···(^δ−^)C─H, C─H(^δ+^)···(^δ−^)π and dispersion interactions. From the independent gradient model based on Hirshfeld partition (IGMH), there are clear blue areas between hydroxy group of AXT/fucoxanthin and μ_3_‐OH of metal cluster correspond to hydrogen bonding (Figure 5b,d). And the green isosurfaces correspond to dispersion interactions are mainly found in β‐carotene incorporated defective UiO‐67 model (Figure 5f).

### 2.5 Desorption Properties of d‐UiO‐67‐X Series for AXT

**Figure 6:**
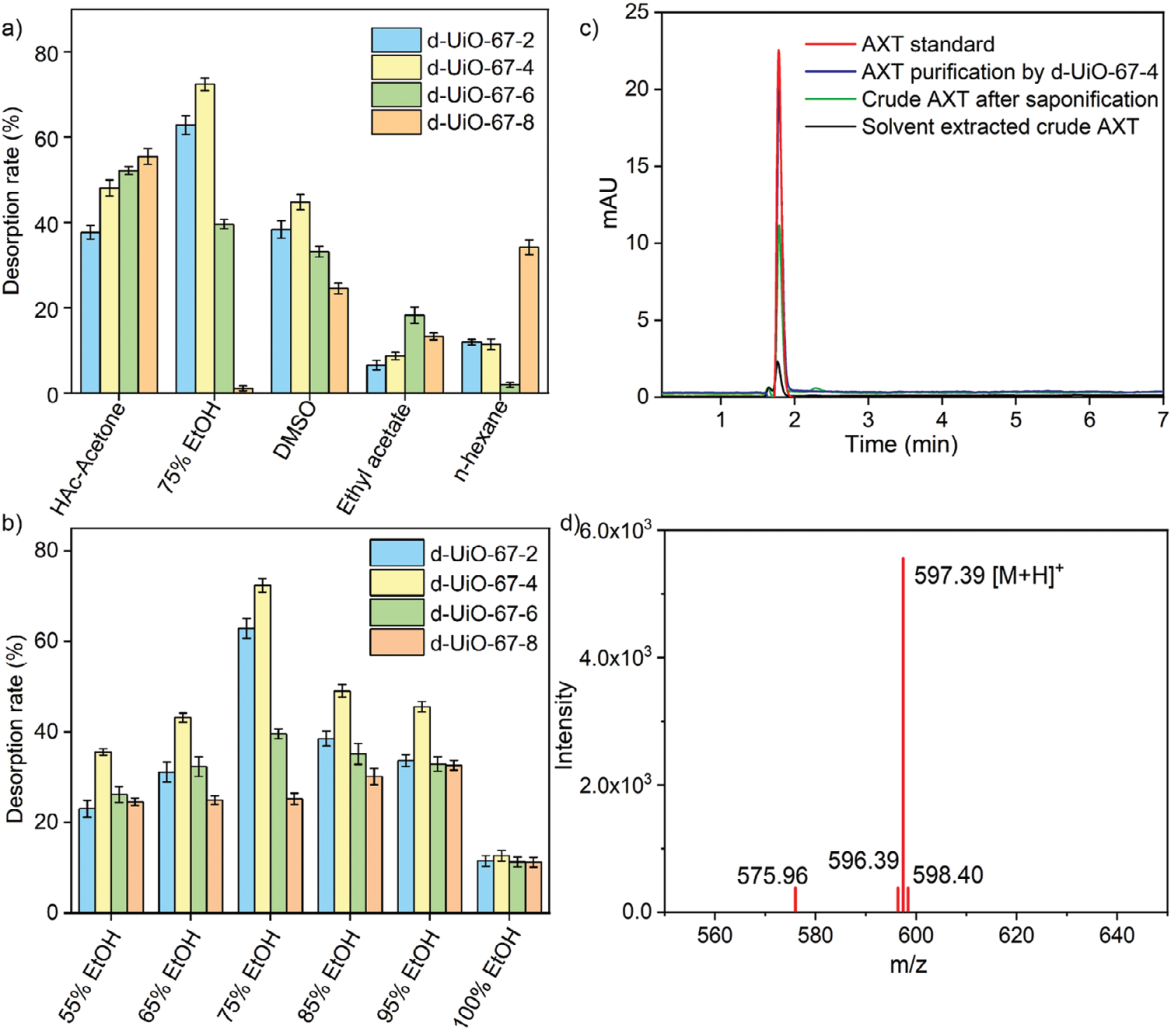
a) Desorption rate of d‐UiO‐67‐X by different eluent solvents. b) Desorption rate of d‐UiO‐67‐X by different ratio of ethanol‐water mixture. c) HPLC spectra of AXT standard, AXT purification by d‐UiO‐67‐X, crude AXT after saponification and solvent extracted crude AXT with the same concentration (4 mg L^−1^).

The desorption performance of AXT from d‐UiO‐67‐X was further determined. The released AXT had the adsorption peak at 480 nm, in agreement with the characteristic adsorption peak of AXT. The release percentage of AXT from d‐UiO‐67‐X was highly affected by elution solvents with different polarities, as shown in Figure 5. The elution solvents including n‐hexane, ethyl acetate, DMSO, ethanol/water and acetic acid/acetone were used to release AXT. As shown in **Figure** **6**a, with the use of 75% ethanol and 25% water mixture, the release percentage of AXT from d‐UiO‐67‐4 was the highest (72.41%) compared to other elution solvents. It should be noted that the release effect was highly affected by water content (0–45%) in ethanol (Figure 6b). When only ethanol uses as elution solvent, the release of AXT was extremely low (≈11.22–12.66%). Using 75% ethanol and 25% water mixture had the higher release percentage of AXT from d‐UiO‐67‐2, d‐UiO‐67‐4 and d‐UiO‐67‐6, compared to other proportion of ethanol‐water mixture. This probably due to the effective disruption of polarity balance and intermolecular interaction between d‐UiO‐67‐4 and AXT by adding 75% ethanol and 25% water, and thus be able to release AXT from d‐UiO‐67‐4.

### 2.6 Separation and Purification of AXT from *Haematococcus pluvialis*

The crude AXT product was obtained from commercial *H. pluvialis* powder by mechanical sonification and solvent extraction, followed by the treatment of saponification process, according to the previous study with some modification.^[^ ^27^ ^]^ Free AXT with the purity of 10.22% was found in *H. pluvialis*. The purity of AXT after saponification was increased to 48.6% by HPLC‐UV analysis. This indicates that many impurities that contains inorganic salts, fatty acids and other extracted carotenoids still present in saponified AXT product.^[^ ^28^ ^]^ The separation and purification performance of AXT by using d‐UiO‐67‐4 adsorbent was evaluated. As shown in Figure 6c, the released AXT after purified by d‐UiO‐67‐4 have an adsorption peak at 480 nm with the retention time of 1.78 min, consistent with the standard AXT HPLC profile (retention time 1.78 min). Meanwhile, the released AXT from d‐UiO‐67‐4 was characterized by atmospheric pressure chemical ionization tandem mass spectrometry (APCI‐MS). As shown in Figure 6d, the detected AXT was found as the ion with a mass to charge ratio (m/z) of 597.39 in positive ionization mode. The purity of the released AXT from d‐UiO‐67‐4 by HPLC‐UV analysis was remarkably increased from the initial 10.22% ± 0.86% to 89.0% ± 2.3% after the purification by d‐UiO‐67‐4, with the ≈8.7 times enhancement in purity. As the above results, d‐UiO‐67‐4 had the great potential for separation and purification of AXT. As shown in Figure S30 (Supporting Information), no significant loss in the adsorption efficiency of AXT (≈87.9%) was found in the 5th regenerated d‐UiO‐67‐4. The PXRD pattern of d‐UiO‐67‐4 after the 5th recycling experiment of adsorption and desorption of AXT (Figure S31, Supporting Information) was still remained, which indicated the structure integrity without structure collapse.

### 2.7 Fixed‐Bed Continuous Adsorption and Desorption Performance

**Figure 7:**
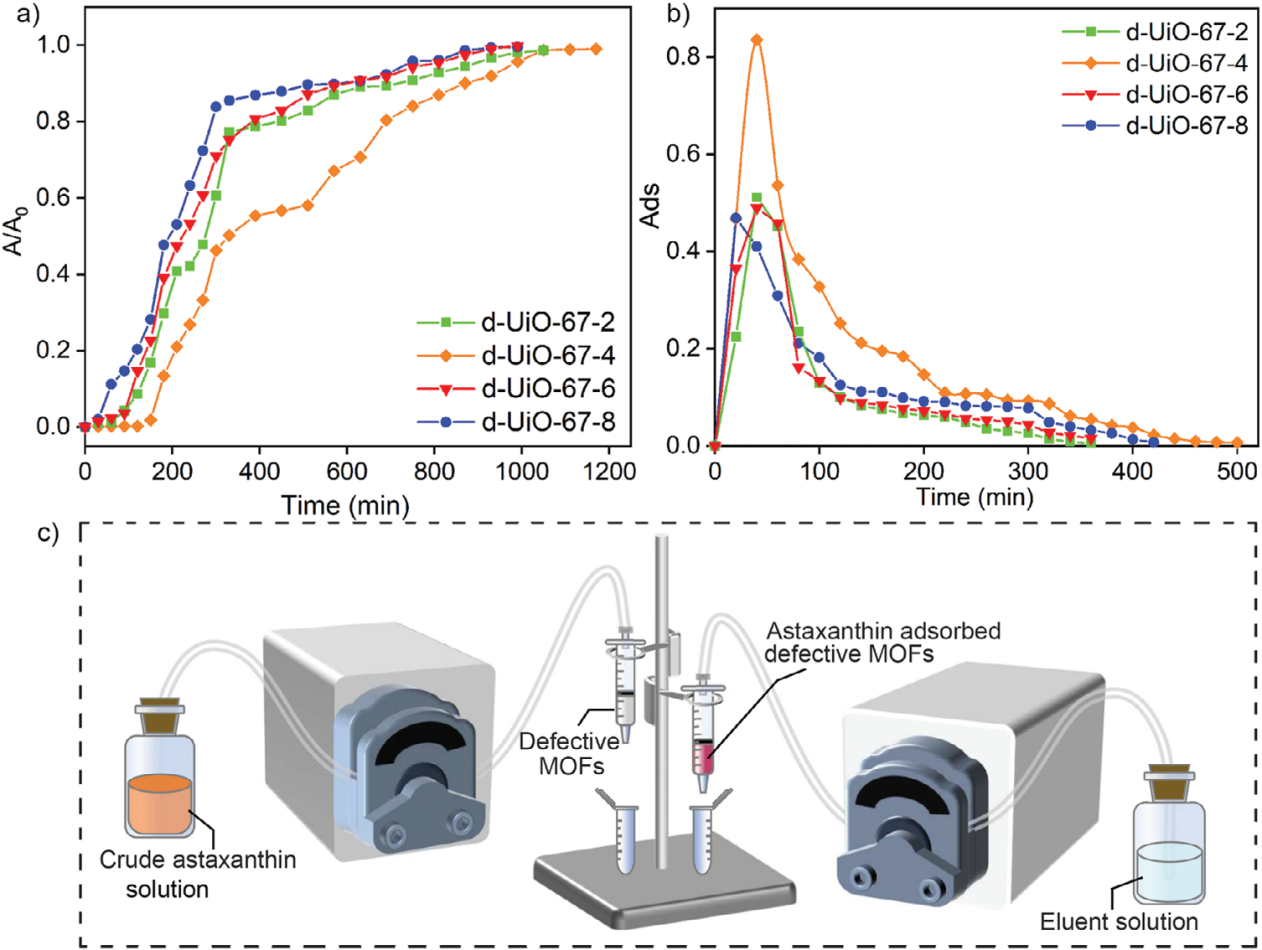
a) Breakthrough curves of fixed‐bed adsorption and b) desorption for d‐UiO‐67‐X and c) the correspond schematic diagram of fixed‐bed column apparatus.

Fixed‐bed column sorption study was carried out by a continuous flow of extracted AXT solution through a fixed height column to estimate the practicality of d‐UiO‐67‐X series for dynamic adsorption and desorption performance of AXT (**Figure** **7**). The breakthrough adsorption curves by plotting A/A_0_ versus adsorption time were shown in Figure 7a. The breakthrough time (*t*_b_) and exhaustion time (*t*_e_) represent the time at that effluent AXT concentration reached 10% and 90% of the initial AXT concentration. The longest *t*_e_ (ca.1050 min) was found in d‐UiO‐67‐4, and exhaustion occurred rapidly in d‐UiO‐67‐8 (ca.870 min). From dynamic desorption curves (Figure 7b), the absorbance of AXT in the eluent increased rapidly at the beginning, and with the increase of desorption time, the desorbed AXT gradually decreased. It can be clearly seen that d‐UiO‐67‐4 had the best desorption capacity per unit volume of eluent. The fixed‐bed column adsorption and desorption results confirmed d‐UiO‐67‐4 as a promising adsorbent for selectively capturing AXT and highly efficient desorption of AXT, which have the great potential for practical application.

## 3 Conclusion

In summary, defective d‐UiO‐67‐X was successfully constructed by modulator‐induced defect engineering strategy, and structure defect was controlled by modulator‐to‐linker ratio in the precursor solution. The pore size and pore chemistry could be tuned through the controlled defects to optimize the AXT separation performance. The d‐UiO‐67‐4 exhibited the high adsorption capacity of 26.21 mg g^−1^ for AXT, which was ≈3.6 times higher than the non‐defective UiO‐67 (7.22 mg g^−1^). Furthermore, d‐UiO‐67‐4 displayed satisfiable adsorption selectivity for AXT over other carotenoids, attributed to the dominated multiple hydrogen bonding coupling with vdW interactions as proven by DFT calculations. The practical potential for the separation performance of AXT extracted from *H. pluvialis* on d‐UiO‐67‐4 was further demonstrated by fixed‐bed column dynamic sorption experiment and the purity of AXT by d‐UiO‐67‐4 can achieve 89.0% ± 2.3%. This study provides a feasible and effective method for selective separation and purification of AXT. This method in our work can be also applicable to other porous materials for selectively separating the target bioactive compound from complex matrix.

## Conflict of Interest

The authors declare no conflict of interest.

## Supporting information



Supporting Information

## Data Availability

The data that support the findings of this study are available from the corresponding author upon reasonable request.
