# Supplementary material for: Fine‐Tuning Porous Structure of Zirconium‐Based Metal–Organic Frameworks for Efficient Separation and Purification of Astaxanthin by Defect Engineering
Source: Adv Sci (Weinh). 2024 Nov 14;11(48):2409451. doi: 10.1002/advs.202409451 (PMC11672258; doi:10.1002/advs.202409451)
Supplement: Supplementary file 1 — Supporting Information [file ADVS-11-2409451-s001.docx]

Supporting Information

Fine-tuning porous structure of zirconium-based metal-organic frameworks for efficient astaxanthin separation by defect engineering

Xin Na, Shanghua Xing*, Wentao Su*, Mingqian Tan

X. Na, S. H. Xing, M. Q. Tan, W. T. Su

State Key Laboratory of Marine Food Processing and Safety Control,

Dalian Polytechnic University, Dalian 116034, Liaoning, China.

E-mail: xingshanghua@126.com; suwentao2020@yeah.net

X. Na, S. H. Xing, M. Q. Tan, W. T. Su
Academy of Food Interdisciplinary Science, School of Food Science and Technology,

Dalian Polytechnic University, Dalian 116034, Liaoning, China.

X. Na, S. H. Xing, M. Q. Tan, W. T. Su
National Engineering Research Center of Seafood,

Dalian Polytechnic University, Dalian 116034, Liaoning, China.

X. Na, S. H. Xing, M. Q. Tan, W. T. Su
Collaborative Innovation Center of Seafood Deep Processing,

Dalian Polytechnic University, Dalian 116034, China.

Experimental Section

*Materials*: Anhydrous zirconium tetrachloride (ZrCl_4_) was purchased from Thermo Fisher Scientific (China) Co., Ltd. AXT with a purity of 96% was obtained from two sources: Shanghai Maikelin Reagent Co., Ltd. and Chengdu PuSi Biotechnology Co., Ltd. Additionally, 4,4'-biphenyl diacetic acid was procured from Shanghai Maikelin Reagent Co., Ltd. Rain-fed chlorella powder was purchased from Yunnan Boxin Runsheng Biotechnology Co., Ltd. Methanol, dimethyl sulfoxide, acetic acid, N,N-dimethylformamide and n-hexane were purchased from Shanghai Maikelin Reagent Co., Ltd. Acetone was obtained from Guangdong Xilang Science Co., Ltd. Ethyl acetate was sourced from Tianjin Kemio Chemical Reagent Co., Ltd. Anhydrous ethanol was acquired from Tianjin Fuyu Fine Chemicals Co., Ltd. Chromatography-grade dichloromethane was procured from Tianjin Damao Chemical Reagent Factory. Chromatography-grade acetonitrile was purchased from Shanghai Maikelin Reagent Co., Ltd. Lycopene (98% purity), β-carotene (≥96% purity), and fucoxanthin (98% purity) were obtained from Shanghai Aladdin Biochemical Technology Co., Ltd. Lutein (90% purity) was acquired from Chengdu PuSi Biotechnology Co., Ltd. A chlorophyll A and B mixture was sourced from Shanghai Maikelin Reagent Co., Ltd.

*Synthesis of UiO-67 and d-UiO-67-X*: UiO-67 [Zr_6_O_4_(OH)_4_(BPDC)_6_] (BPDC = 4,4'-biphenyl dicarboxylate) was synthesized by solvothermal method according to the reference.^[1]^ ZrCl4 (52.9 mg, 0.227 mmol) and BPDC (55 mg, 0.227 mmol) were dissolved in 5 mL of N,N-dimethylformamide (DMF) using ultrasonication and subsequently stirred for 2 h. The resulting mixture was then transferred to a Schott vial, sealed, and heated at 120 °C for 24 h. Upon cooling to room temperature, the precipitate was collected by centrifugation and washed thrice with DMF. Finally, the product was dried overnight under vacuum at 70 °C. To prepare the defective d-UiO-67-X (X = 2, 4, 6 and 8), different amounts of acetate acid as modulator was incorporated during the synthesis of UiO-67 to partially replace the BPDC linker in structure. ZrCl4 (52.9 mg, 0.227 mmol) was combined with varying molar ratios of acetic acid to BPDC (2:1, 4:1, 6:1, and 8:1) and dissolved in 5 mL of DMF. The mixture was sonicated until a clear solution was obtained. The resulting solution was then sealed in a Schott vial and heated at 120 °C for 24 hours. Upon cooling to room temperature, the precipitate was collected by centrifugation and washed with DMF (3 × 5 mL). Finally, the product was dried overnight under vacuum at 70 °C.

*Characterization*: X-ray diffraction (XRD) patterns were collected using an XRD-6100 diffractometer over the 2θ range of 5-50° under operating conditions of 30 kV and 20 mA. Fourier transform infrared (FT-IR) spectra were recorded on a Spectrum Two spectrometer (PerkinElmer, Japan) in the range of 400-4000 cm⁻¹. ¹H NMR spectra were acquired using a Bruker AVANCE III 400 MHz spectrometer. Sample preparation for NMR analysis involved digesting 18 mg of sample with 23 mg NaOH in 0.55 mL D_2_O_2_ followed by sonication to obtain a clear solution. Morphological characterization was performed using a JSM-7800F scanning electron microscope (SEM) at an accelerating voltage of 5 kV and a JEM-2100(UHR) transmission electron microscope (TEM). Energy-dispersive X-ray spectroscopy (EDS) was conducted using an X-Max 50 spectrometer. Brunauer-Emmett-Teller (BET) surface area measurements were carried out on a Micromeritics ASAP 2460 analyzer. Thermogravimetric analysis (TGA) was performed on a TGA-550 analyzer from 30 to 800 °C at a heating rate of 10 °C min⁻¹ under a nitrogen atmosphere. X-ray photoelectron spectroscopy (XPS) was conducted using a Thermo Fisher Nexsa G2 instrument to elucidate chemical states, surface properties, and composition. Astaxanthin (AXT) concentration was determined using a SP-2500 UV-visible spectrophotometer in the range of 350-700 nm, with acetone as the blank. High-resolution mass spectrometry of purified AXT was performed on a Thermo Scientific Q Exactive Orbitrap mass spectrometer equipped with an atmospheric pressure chemical ionization (APCI) source.

*Adsorption measurement*: Prior to adsorption experiment, UiO-67 and d-UiO-67-X samples were vacuum-dried overnight at 150 ℃. All adsorption experiments were conducted in AXT solutions at ambient temperature (25 ± 2°C) under light-protected conditions. In a typical adsorption assay, 10 mg of UiO-67 or d-UiO-67-X was introduced into 5 mL of AXT solution at concentrations of 2, 4, or 8 mg L^-1^. At predetermined time intervals, aliquots of the supernatant were withdrawn and analyzed using UV-VIS spectrophotometry. Following spectral analysis, the supernatant was immediately returned to the reaction vessel to maintain constant volume. The adsorption capacity (*q_t_*, mg g^-1^) at time *t* was calculated using the following equation: ^[2]^

$q_{t}=\frac{(C_{0}-C_{t})V}{m}$ (1)
$q_{e}=\frac{(C_{0}-C_{e})V}{m}$ (2)

where *C_0_*, *C_t_* and *C_e_* represent the AXT concentrations (mg L⁻¹) in solution at the initial stage, at time *t*, and at equilibrium, respectively. *V* denotes the volume of the solution (L), and *m* represents the mass of the UiO-67/d-UiO-67-X adsorbent (g).

*Adsorption kinetics*: The time-dependent adsorption capacity of d-UiO-67 for AXT solutions with initial concentrations of 2, 4, and 8 mg L^-1^ was analyzed using linear pseudo-first-order and pseudo-second-order kinetic models. These models are represented by the following equations: ^[3]^

$ln(q_{e}-q_{t})=\ln q_{e}-k_{1}t$ (3)

$\frac{t}{q_{t}}=\frac{1}{k_{2}{q_{e}}^{2}}+\frac{t}{q_{e}}$ (4)

The adsorption kinetics were analyzed using pseudo-first-order and pseudo-second-order models. In these models, *q_t_* and *q_e_* (mg g^−1^) represent the quantity of anthocyanin adsorbed at time *t* and at equilibrium, respectively. The rate constants for the pseudo-first-order and pseudo-second-order models are denoted as *k_1_* (min^−1^) and *k_2_* (g mg^−1^ min^−1^), respectively.

The intraparticle diffusion kinetic models is using the following equation:^[4]^

$q_{t}=k_{i}t^{0.5}+C_{i}$ (5)

Where *k_i_* is the constant of the intraparticle diffusion kinetic adsorption (nmol g^-1^ min^0.5^), and *C_i_* is the intercept of linear curve.

*Adsorption isotherms*: Adsorption experiments were conducted using 10 mg of d-UiO-67-X to adsorb 5 mL of AXT solution with concentrations ranging from 2 to 56 mg L^−1^. The adsorption isotherms were analyzed using both linear Langmuir and Freundlich isotherm models. These models were applied using the following equations: ^[5]^

$\frac{C_{e}}{q_{e}}=\frac{1}{K_{L}Q_{m}}+\frac{C_{e}}{Q_{m}}$ (6)

$lnq_{e}=lnK_{F}+\frac{1}{n}lnC_{e}$ (7)

Where *K_L_* and *K_F_* represent the Langmuir and Freundlich constants, respectively; *q_e_* (mg g^−1^) denotes the equilibrium adsorption capacity; *C_e_* (mg L^−1^) signifies the equilibrium concentration of the AXT solution; *Q_m_* (mg g^−1^) indicates the maximum adsorption capacity; and 1/n is a dimensionless empirical constant that reflects the intensity of the adsorption process.

*Desorption experiment*: In a typical desorption experiment, 10 mg of AXT adsorbed UiO-67/d-UiO-67-X was added in 5 mL of elution solution at room temperature and by avoiding the light. The 4% HAc-Acetone, 55%-95% ethanol in water, anhydrous ethanol, DMSO, ethyl acetate and n-hexane solution were used as the elution solvent. After desorption completion, UV-VIS absorption spectra of released AXT solution from UiO-67/d-UiO-67-X were measured after centrifugation. Each desorption experiment was repeated for three times. The desorption rate was calculated by the following equation:

$Desorption rate \left( \% \right)=\frac{C_{d}}{C_{0}}\times100\%$ (8)

Where *C_0_* and *C_d_* (mg L^−1^) are the initial AXT concentration and the desorbed AXT concentration, respectively.

*Extraction of crude AXT*: AXT was extracted by solvent extraction. *Haematococcus pluvialis* powder (500 mg) was placed in a beaker with 10 mL dichloromethane. After the mixture was evenly mixed, ultrasonic cell crusher was used for ultrasonic crushing for 5 min at 500 W. Then it was heated and stirred at 50 ℃, and the extraction time was 40 min. After the supernatant was removed by centrifugation, a new extraction solution was added and the extraction process was repeated with 4 times until the light color of filtrated solution and then freeze drying.^[6]^

*Saponification of extracted crude AXT*: The freeze-dried crude AXT extract was weighed and added to anhydrous ethanol for ultrasonic dissolution, followed by centrifugation to remove impurities. The crude AXT solution was adjusted the pH to 10 by adding 10% KOH-ethanol solution, and adjusted the pH to neutral by adding 10% diluted hydrochloric acid after 2.5 h saponification in the refrigerator at 4 ℃. Finally, the de-esterified AXT powder was obtained after freeze drying.^[7]^

*Purification of AXT by d-UiO-67-4*: 1 g of activated d-UiO-67-4 was added into 10 mL ethanol solution of de-esterified AXT with the concentration of 20 mg L^-1^ and stirred at room temperature under a speed of 100 r min^-1^. After adsorption equilibrium was reached, the solution was filtered to separate the adsorbent. And then the separated adsorbent was washed by 10 mL of deionized water and 10 mL of ethanol, and then desorbed by adding 10 mL of 60% ethyl acetate-ethanol elution solution at 30 °C and stirred at 100 r min^-1^ for 12 h. The filtrate was collected and evaporated at 30 °C followed by freeze drying to obtain the purified AXT powder.

The purified astaxanthin (AXT) was analyzed using an Agilent 1260 Infinity II High-Performance Liquid Chromatography (HPLC) system equipped with a UV detector set at a wavelength of 480 nm. Chromatographic separation was achieved using an Elite Hypersil C18 column (250 mm × 4.6 mm, 5 μm particle size). The mobile phase consisted of two solvents: Solvent A (dichloromethane/methanol/acetonitrile/water, 5.0:85.0:5.5:4.5, v/v) and Solvent B (dichloromethane/methanol/acetonitrile/water, 22.0:28.0:45.5:4.5, v/v). The flow rate was maintained at 1.0 mL min^−1^, and the injection volume was 20 μL. A gradient elution program was employed as follows: 100% Solvent A was maintained for 8 minutes, followed by a linear gradient from 0% to 100% Solvent B over 24 minutes. ^[8]^

The HPLC peak area for evaluating the purity of AXT was calculated by the following equation:

$Purity \left( \% \right)=\frac{S_{1}}{S_{0}}\times100\%$ (9)

Where *S_0_* and *S_1_* are the peak areas of standard AXT and purified AXT/de-esterified AXT solution. Note that the standard AXT was prepared with the same concentration (4 mg L^−1^) of purified AXT/de-esterified AXT solution.

*Fixed-bed column adsorption and desorption experiment*: 200 mg of activated d-UiO-67-X was added into the EZ gravity column (d = 9.56 mm and l = 55 mm) and then rinsed with 20 mL ethanol through a peristaltic pump. For the adsorption process, 30 mg L^−1^ of saponified AXT solution was pressed through the chromatographic column by a peristaltic pump at a flow rate of 0.25 mL min^−1^. After the adsorption, AXT adsorbed in column was eluted with 60% ethyl acetate-ethanol solution at a flow rate of 0.5 mL min^−1^. During the adsorption and desorption process, 5 mL of effluent was collected periodically and determined by at 480 nm by UV-VIS spectroscopy.^[9]^

*DFT calculations*: All density functional theory (DFT) calculations were conducted using the Becke, three-parameter, Lee-Yang-Parr (B3LYP) hybrid functional as implemented in the Gaussian 16 software package. ^[10]^ The cluster models were isolated from the crystal structure of UiO-67 and use as the initial configuration of geometry optimization by B3LYP-D3 functional.^[11]^ The double-ζ basis set LANL2DZ was used for Zr atoms, and the 6-311G** basis set was used for the other elements. To mimic the defect structure environment induced by acetate acid modulator, one acetate was replaced the position of the original BPDC ligand. In geometry optimization, the positions of the Zr atoms were fixed to those in the experimental structures. By referring the other reported MOF-based cluster models,^[12]^ the bridging BPDC ligands around a Zr_6_ cluster in UiO-67 was replaced by formate ligands to ensure the electroneutrality of the cluster model.


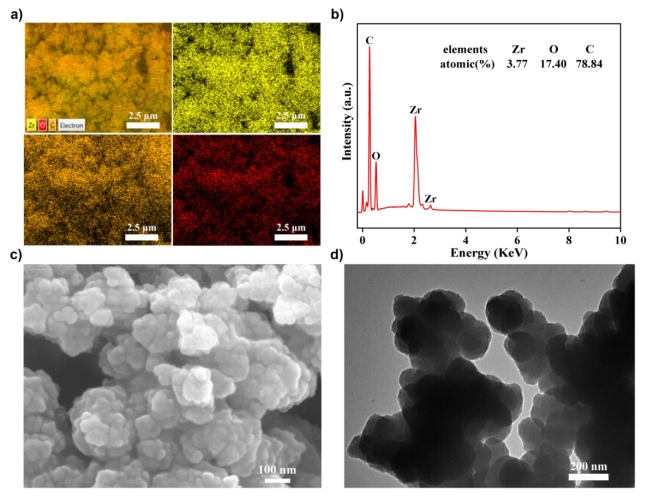


Figure S1.EDS maps (a) and spectrum (b) of the detected elements in the analyzed region of the SEM image for UiO-67; SEM images (c) and TEM images (d) of UiO-67.


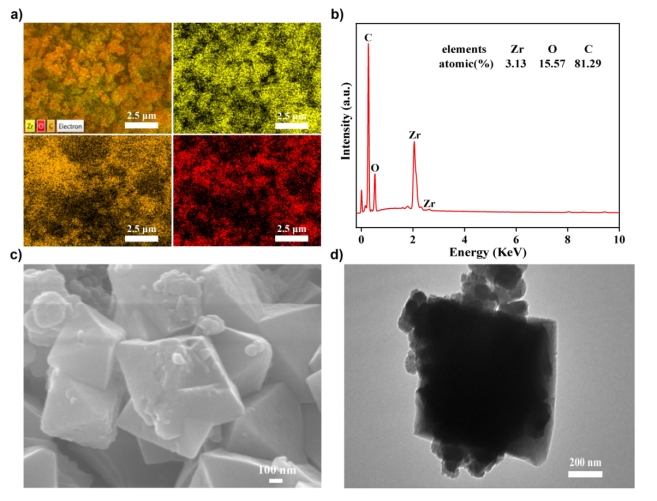


Figure S2.EDS maps (a) and spectrum (b) of the detected elements in the analyzed region of the SEM image for d-UiO-67-2; SEM images (c) and TEM images (d) of d-UiO-67-2.


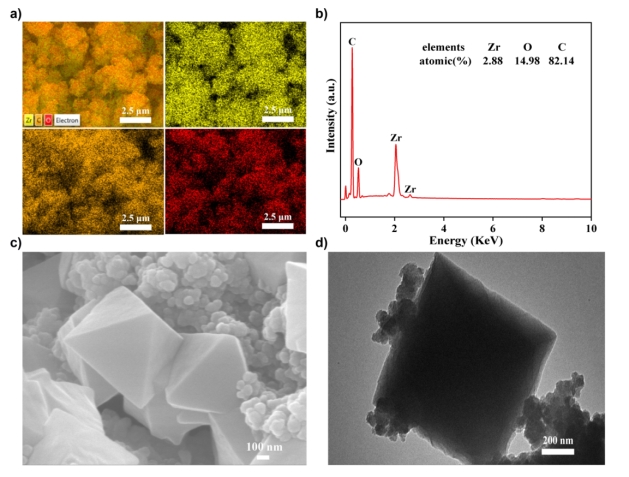


Figure S3.EDS maps (a) and spectrum (b) of the detected elements in the analyzed region of the SEM image for d-UiO-67-4; SEM images (c) and TEM images (d) of d-UiO-67-4.


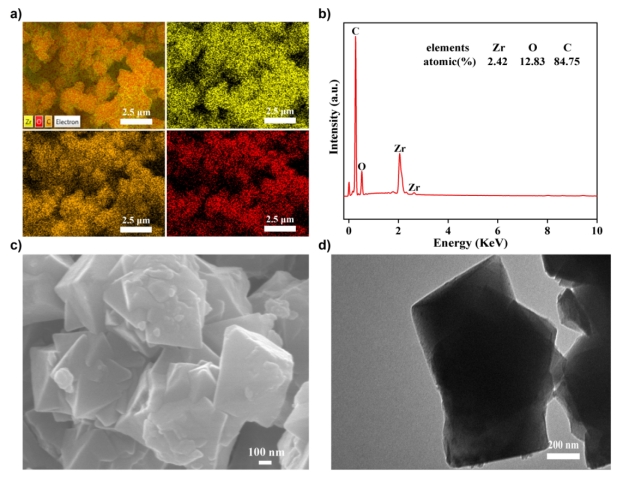


Figure S4.EDS maps (a) and spectrum (b) of the detected elements in the analyzed region of the SEM image for d-UiO-67-6; SEM images (c) and TEM images (d) of d-UiO-67-6.


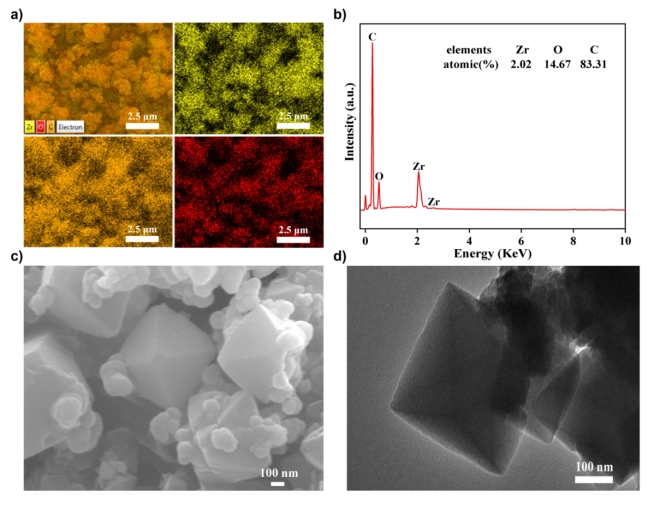


Figure S5.EDS maps (a) and spectrum (b) of the detected elements in the analyzed region of the SEM image for d-UiO-67-8; SEM images (c) and TEM images (d) of d-UiO-67-8.


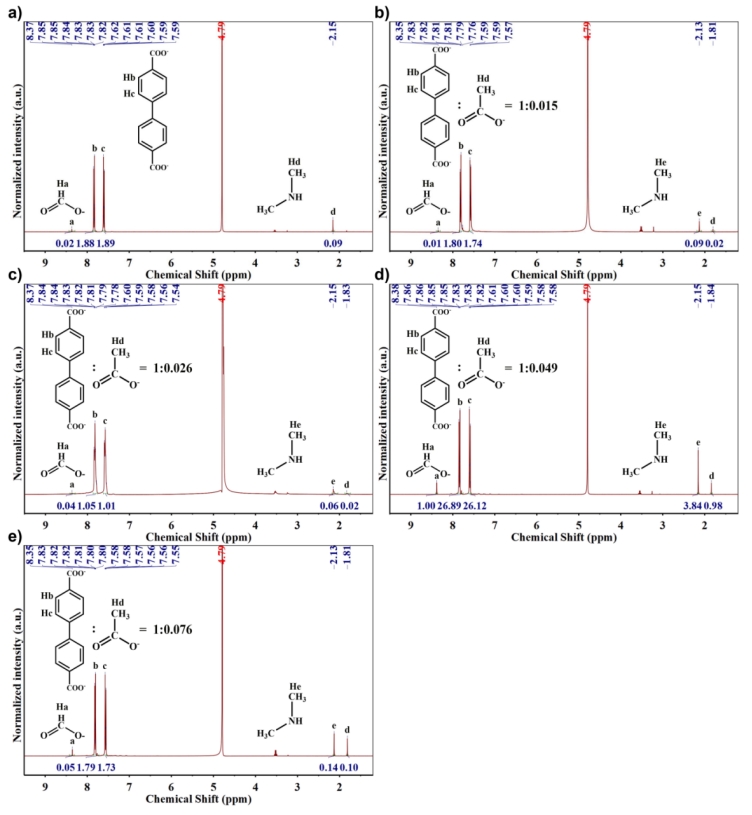


Figure S6. Solution ^1^H NMR spectra of digested UiO-67 (a), d-UiO-67-2 (b), d-UiO-67-4 (c), d-UiO-67-6 (d) and d-UiO-67-8 (e) in NaOH and D_2_O (4.79 ppm). The peaks at ~8.35 and ~1.81 ppm indicates the decomposition of DMF solvent which was also found in other report.^[13]^ The integral ratio of H on BPDC (C_12_H_8_(CO_2_^-^)_2_) and H on acetate (CH_3_COO^-^) in d-UiO-67-2, d-UiO-67-4, d-UiO-67-6 and d-UiO-67-8 appeared to be 3.54:0.02, 2.06:0.02, 53.01:0.98, 3.52:0.1, respectively and thus the molar ratio of BPDC/acetate calculated to be 3.54/8:0.02/3= 1:0.015, 2.06/8:0.02/3=1:0.026, 53.01/8:0.98/3=1:0.049, 3.52/8:0.1/3=1:0.076.


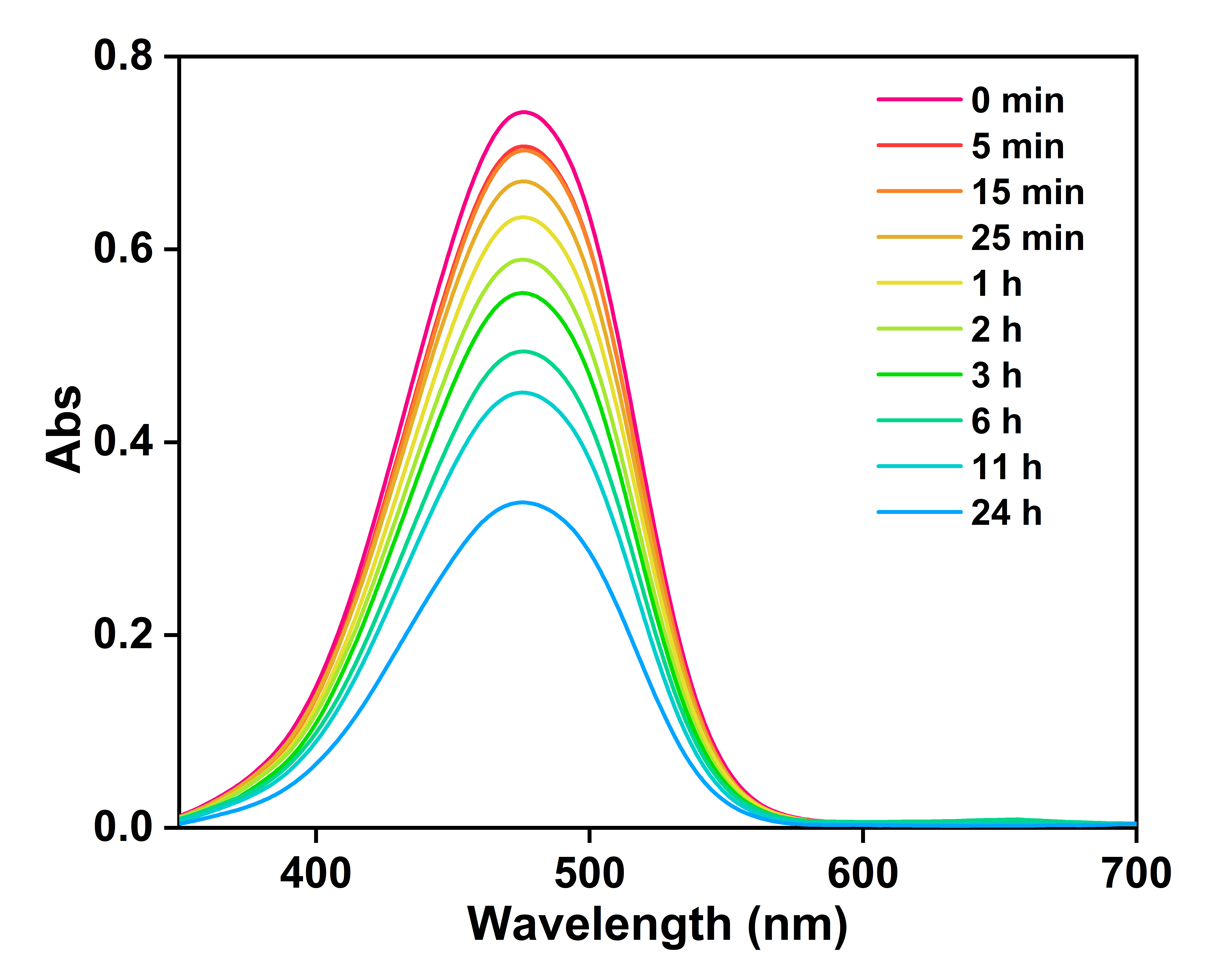


Figure S7. UV-vis spectra of astaxanthin solution (4 mg L^-1^) with different adsorption time for UiO-67.


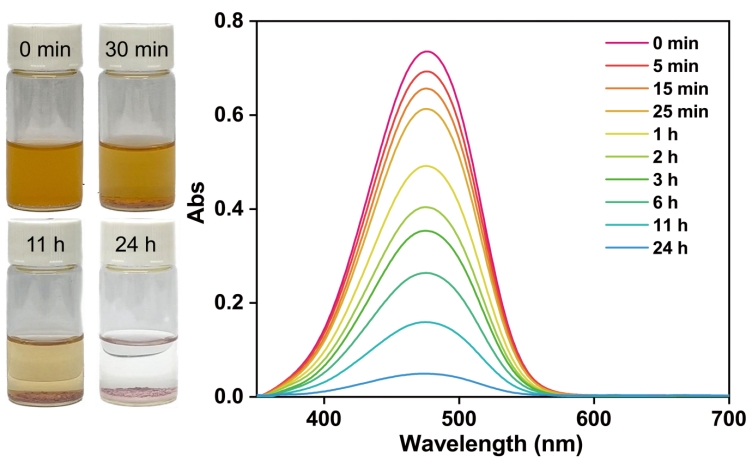


Figure S8. UV-vis spectra of astaxanthin solution (4 mg L^-1^) with different adsorption time for d-UiO-67-2.


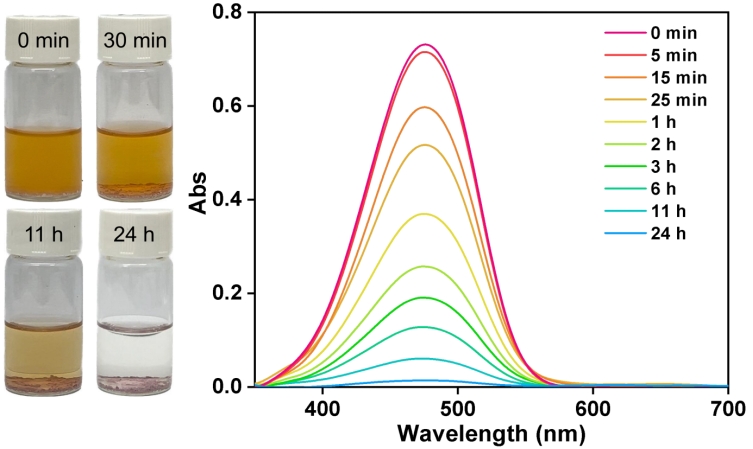


Figure S9.UV-vis spectra of astaxanthin solution (4 mg L^-1^) with different adsorption time for d-UiO-67-4.


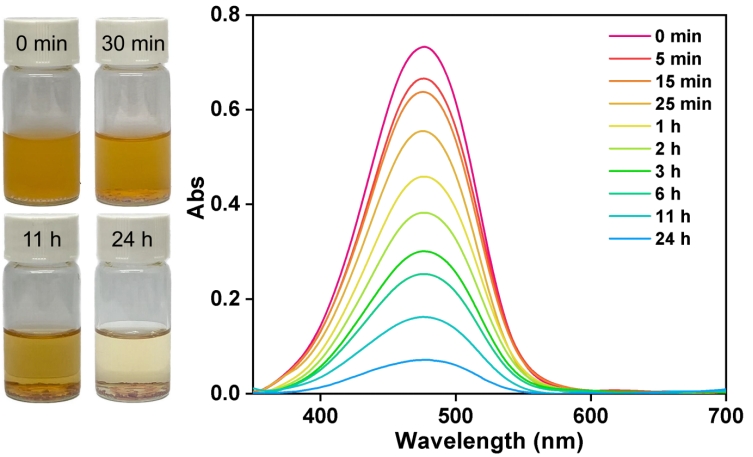


Figure S10.UV-vis spectra of astaxanthin solution (4 mg L^-1^) with different adsorption time for d-UiO-67-6.


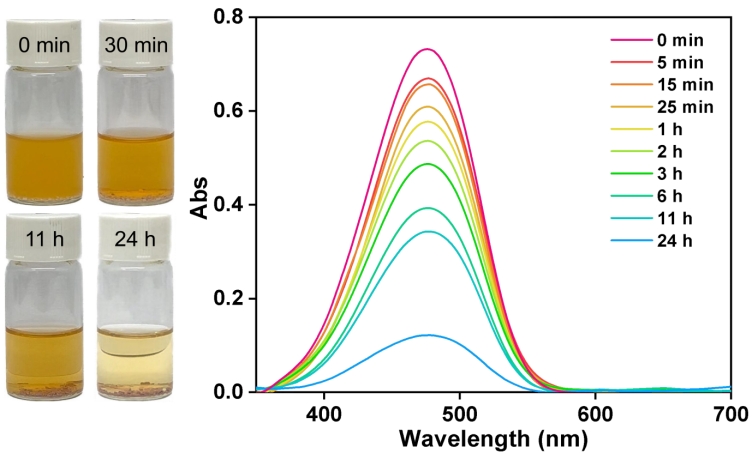


Figure S11.UV-vis spectra of astaxanthin solution (4 mg L^-1^) with different adsorption time for d-UiO-67-8.


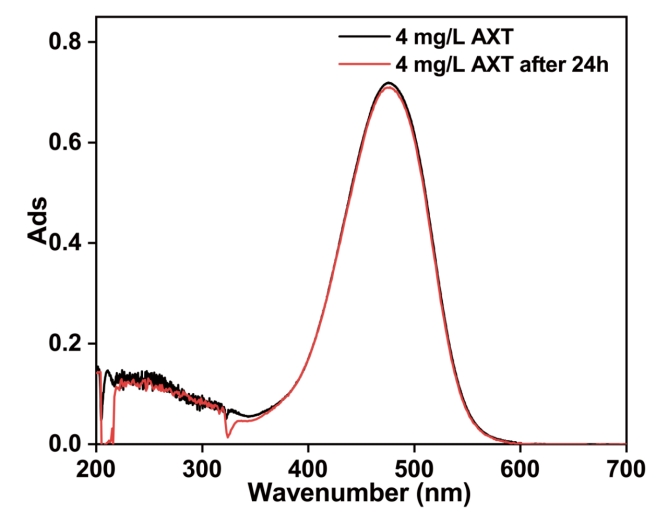


Figure S12. UV-vis absorption spectra of initial astaxanthin solution and after standing for 24 h.


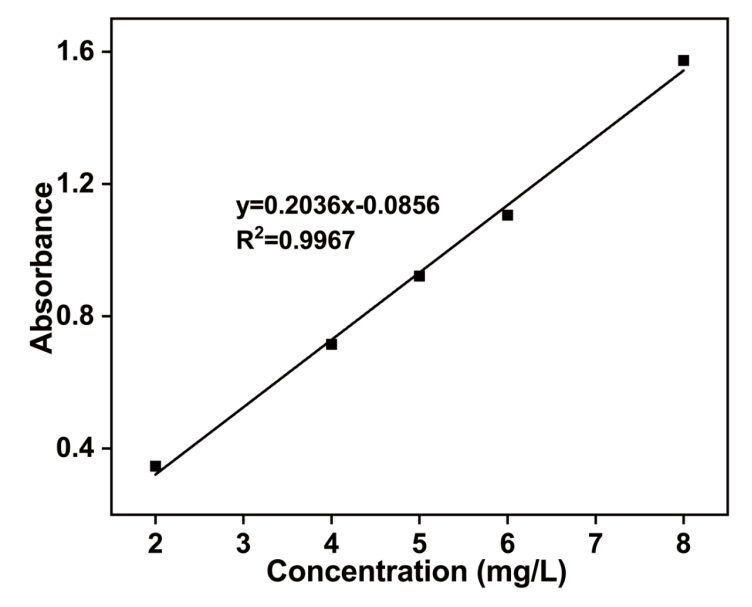


Figure S13. Standard curve for the determination of astaxanthin solution with different concentrations (2-8 mg L^-1^) by UV-vis absorption spectrometry.


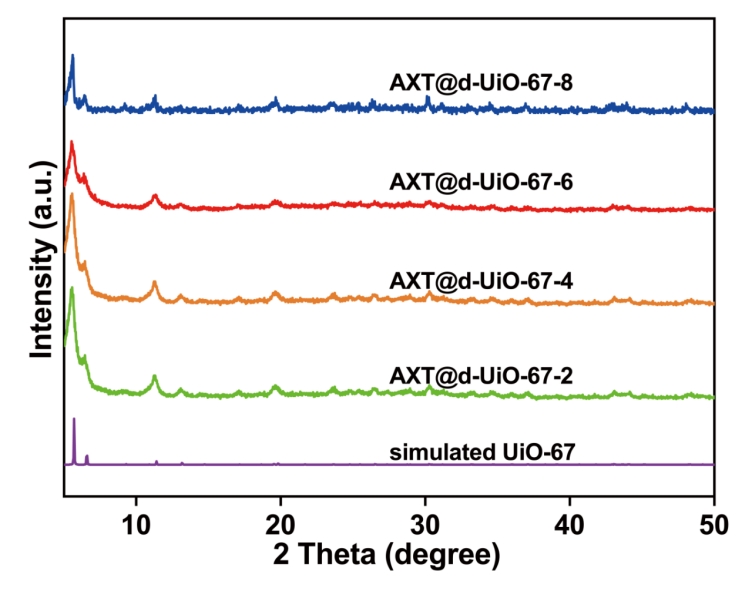


Figure S14.PXRD patterns of astaxanthin adsorbed by UiO-67 and d-UiO-67-X. The simulated PXRD pattern of UiO-67 was obtained from X-ray crystal structure data (CCDC: 2179856).^[14]^


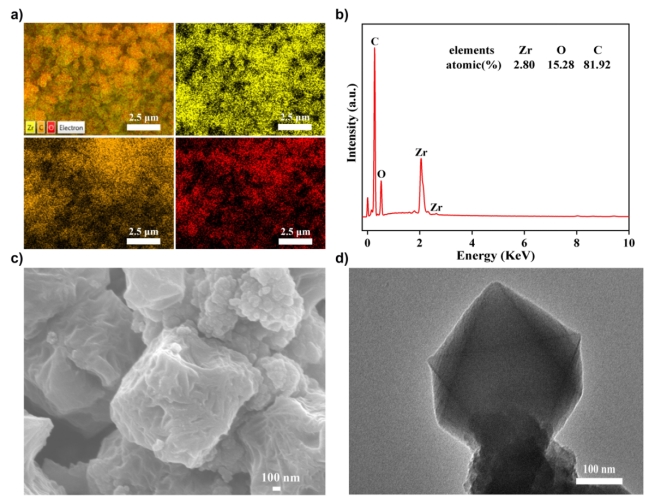


Figure S15.EDS maps (a) and spectrum (b) of the detected elements in the analyzed region of the SEM image for AXT@d-UiO-67-2; SEM images (c) and TEM images (d) of AXT@d-UiO-67-2.


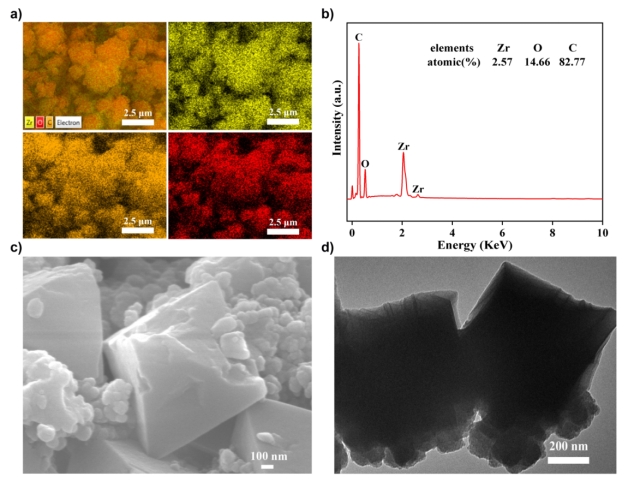


Figure S16.EDS maps (a) and spectrum (b) of the detected elements in the analyzed region of the SEM image for AXT@d-UiO-67-4; SEM images (c) and TEM images (d) of AXT@d-UiO-67-4.


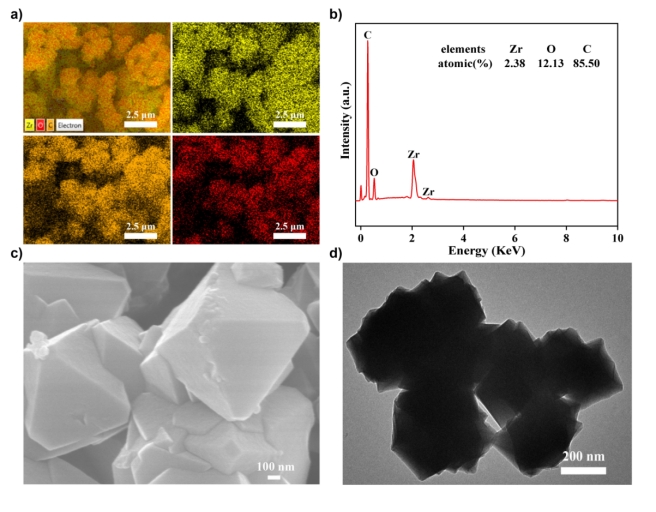


Figure S17.EDS maps (a) and spectrum (b) of the detected elements in the analyzed region of the SEM image for AXT@d-UiO-67-6; SEM images (c) and TEM images (d) of AXT@d-UiO-67-6.


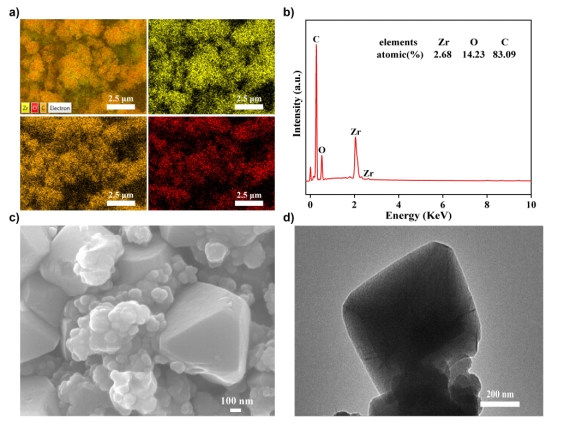


Figure S18.EDS maps (a) and spectrum (b) of the detected elements in the analyzed region of the SEM image for AXT@d-UiO-67-8; SEM images (c) and TEM images (d) of AXT@d-UiO-67-8.


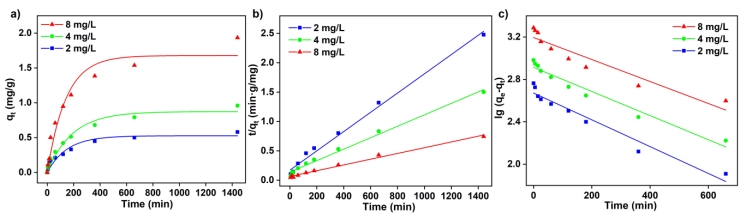


Figure S19.Adsorption capacity of UiO-67 (a) as a function of time with different initial concentrations of astaxanthin. Pseudo-first order kinetic model for astaxanthin adsorption on UiO-67 (b). Pseudo-second order kinetic model for astaxanthin adsorption on UiO-67 (c).


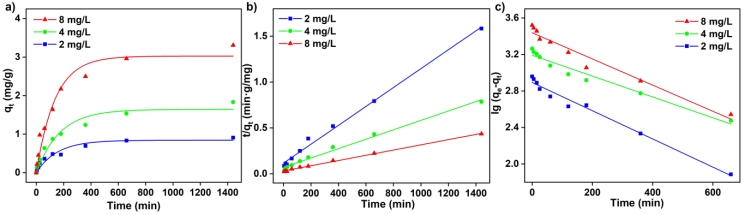


Figure S20.Adsorption capacity of d-UiO-67-2 (a) as a function of time with different initial concentrations of astaxanthin. Pseudo-first order kinetic model for astaxanthin adsorption on d-UiO-67-2 (b). Pseudo-second order kinetic model for astaxanthin adsorption on d-UiO-67-2 (c).


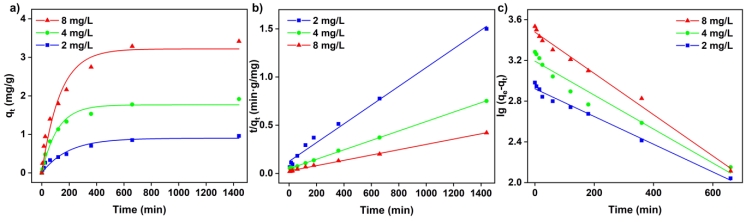


Figure S21.Adsorption capacity of d-UiO-67-4 (a) as a function of time with different initial concentrations of astaxanthin. Pseudo-first order kinetic model for astaxanthin adsorption on d-UiO-67-4 (b). Pseudo-second order kinetic model for astaxanthin adsorption on d-UiO-67-4 (c).


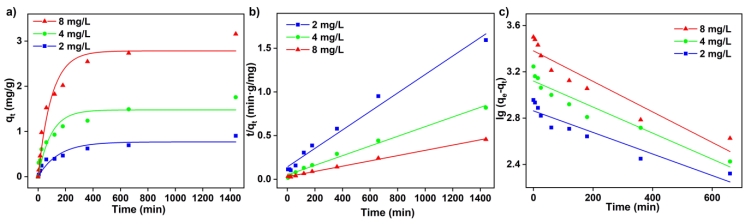


Figure S22.Adsorption capacity of d-UiO-67-6 (a) as a function of time with different initial concentrations of astaxanthin. Pseudo-first order kinetic model for astaxanthin adsorption on d-UiO-67-6 (b). Pseudo-second order kinetic model for astaxanthin adsorption on d-UiO-67-6 (c).


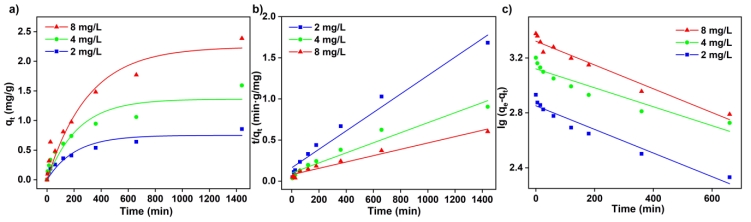


Figure S23.Adsorption capacity of d-UiO-67-8 (a) as a function of time with different initial concentrations of astaxanthin. Pseudo-first order kinetic model for astaxanthin adsorption on d-UiO-67-8 (b). Pseudo-second order kinetic model for astaxanthin adsorption on d-UiO-67-8 (c).


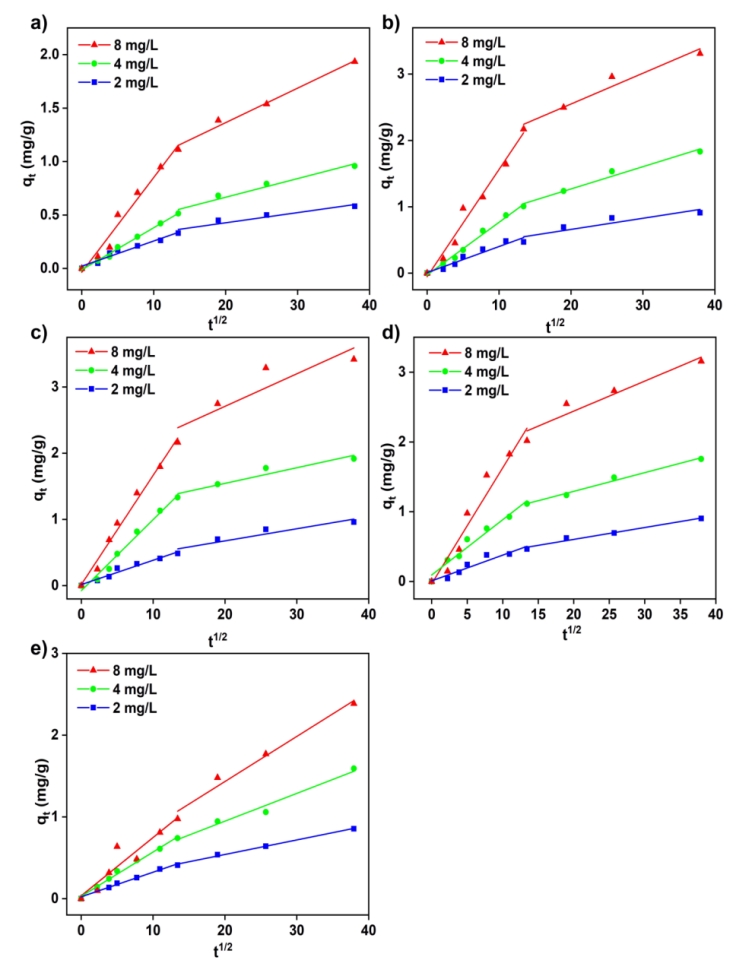


Figure S24.The internal diffusion model of astaxanthin adsorbed by UiO-67 (a), d-UiO-67-2 (b), d-UiO-67-4 (c), d-UiO-67-6 (d) and d-UiO-67-8 (e).


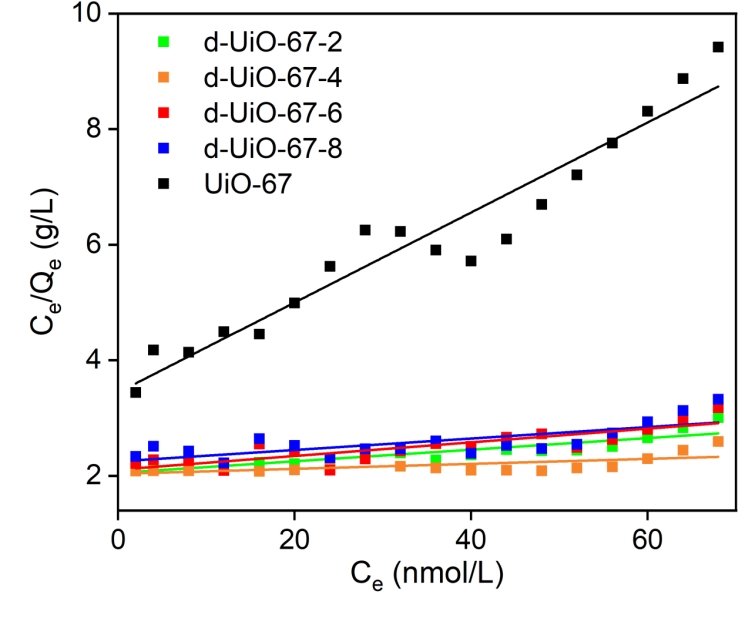


Figure S25. The Langmuir isotherm fitting of UiO-67 and d-UiO-67-X


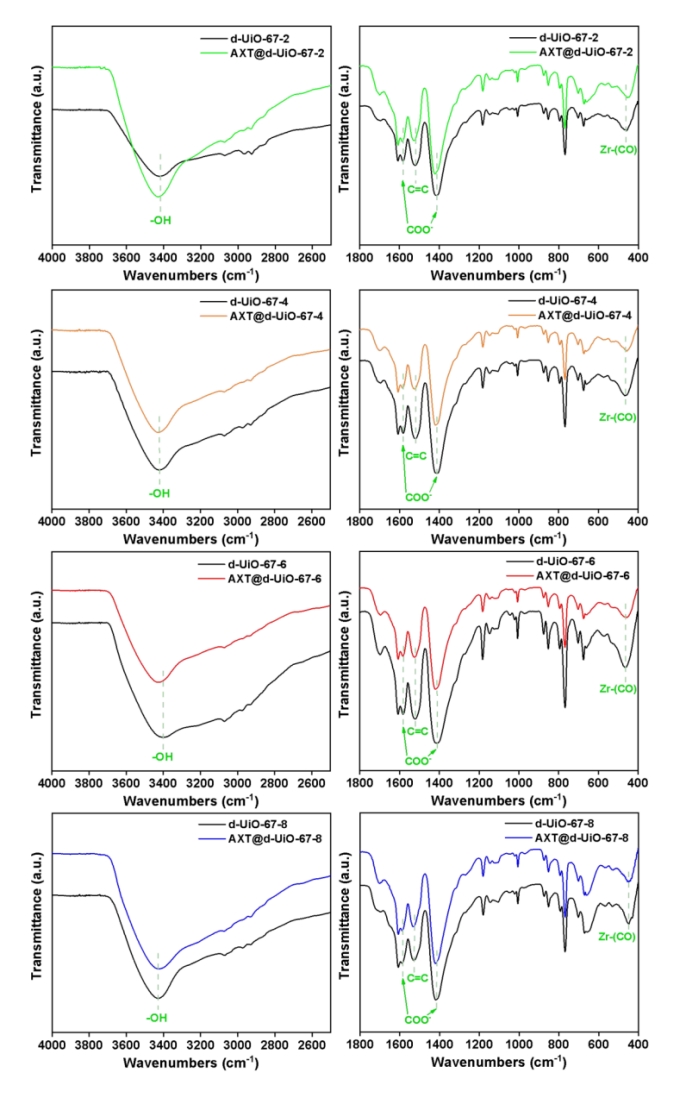


Figure S26. FT-IR spectra of d-UiO-67-X and AXT@d-UiO-67-X.


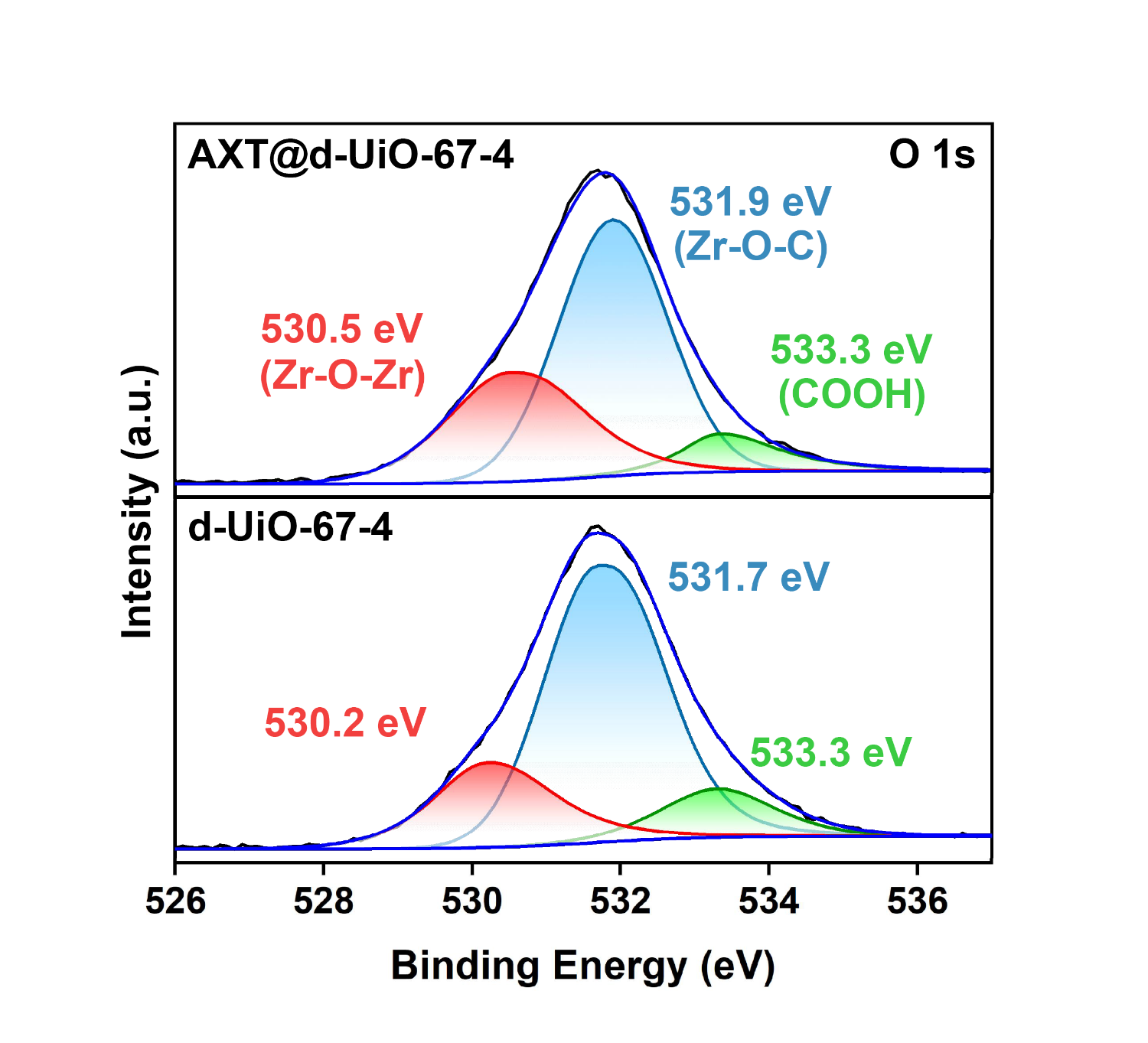


Figure S27. High resolution O1s XPS spectrum of d-UiO-67-4 and AXT@d-UiO-67-4.


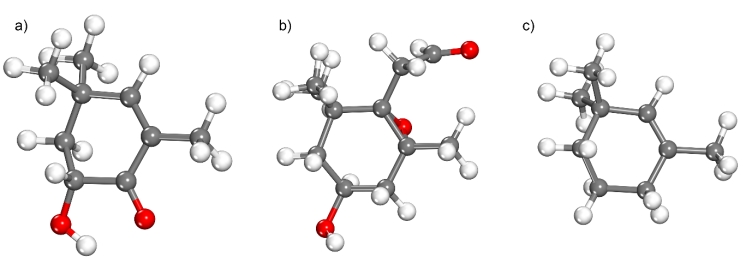


Figure S28. The terminal ring DFT models of AXT (a), fucoxanthin (b) and β-carotene (c)


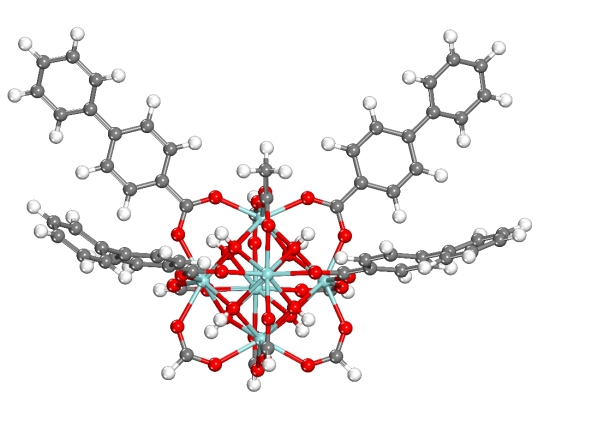


Figure S29. The cluster model of defect UiO-67

**
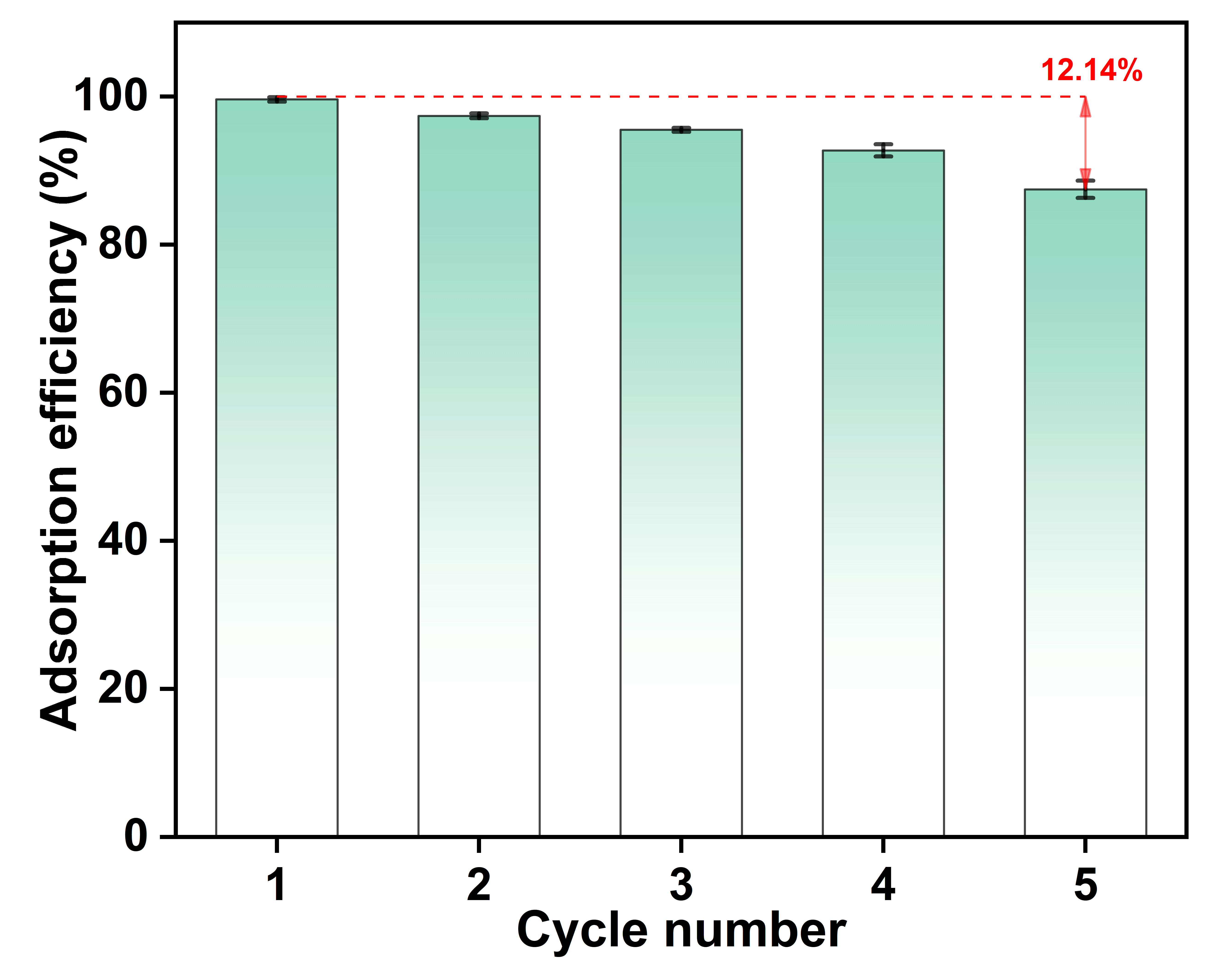
**

Figure S30. Adsorption efficiency of AXT on d-UiO-67-4 for adsorption-desorption recycling experiments.

Figure S31. PXRD pattern of d-UiO-67-4 after 5^th^ recycling experiment of adsorption-desorption processes of AXT.

Table S1. Porosity characteristics of the d-UiO-67-X

| Samples | S_BET_(m^2^ g^-1^) | S_Langmuir_(m^2^ g^-1^) | V_micro_(cm^3^ g^-1^) | V_meso_(cm^3^ g^-1^) | V_total_(cm^3^ g^-1^) | V_micro_/V_meso_ |
| --- | --- | --- | --- | --- | --- | --- |
| UiO-67 | 503.8 | 567.8 | 0.189 | 0.041 | 0.230 | 4.609 |
| d-UiO-67-2 | 674.5 | 904.5 | 0.222 | 0.144 | 0.366 | 1.547 |
| d-UiO-67-4 | 1,153.0 | 1,399.3 | 0.394 | 0.151 | 0.545 | 2.617 |
| d-UiO-67-6 | 1,206.3 | 1,480.0 | 0.421 | 0.155 | 0.576 | 2.715 |
| d-UiO-67-8 | 1,364.1 | 1,631.0 | 0.477 | 0.154 | 0.631 | 3.096 |

Table S2. Adsorption kinetic parameters of astaxanthin on UiO-67

|  | Pseudo-first-order kinetic model | | |  | Pseudo-second-order kinetic model | | |  |
| --- | --- | --- | --- | --- | --- | --- | --- | --- |
| C_0_ (mg L^-1^) | *k*_1_ (min^-1^) | *q*_e_ (mg g^-1^), cal | R^2^ |  | *k*_2_ (g mg^-1^ min^-1^) | *q*_e_ (mg g^-1^), cal | R^2^ | *q*_e_, exp |
| 2 | 0.0041 | 0.5460 | 0.9728 |  | 14.9533 | 0.6250 | 0.9913 | 0.5810 |
| 4 | 0.0044 | 1.0280 | 0.9549 |  | 7.2569 | 1.0000 | 0.9911 | 0.9583 |
| 8 | 0.0048 | 2.1174 | 0.932 |  | 3.9124 | 2.0000 | 0.9905 | 1.9345 |

Table S3. Adsorption kinetic parameters of astaxanthin on d-UiO-67-2.

|  | Pseudo-first-order kinetic model | | |  | Pseudo-second-order kinetic model | | |  |
| --- | --- | --- | --- | --- | --- | --- | --- | --- |
| C_0_ (mg L^-1^) | *k*_1_ (min^-1^) | *q*_e_ (mg g^-1^), cal | R^2^ |  | *k*_2_ (g mg^-1^ min^-1^) | *q*_e_ (mg g^-1^), cal | R^2^ | *q*_e_, exp |
| 2 | 0.0046 | 0.8792 | 0.9859 |  | 8.6505 | 1.0000 | 0.9939 | 0.9094 |
| 4 | 0.0048 | 2.0427 | 0.9457 |  | 3.6284 | 2.0000 | 0.9909 | 1.8329 |
| 8 | 0.0053 | 3.4930 | 0.9616 |  | 2.9221 | 3.3333 | 0.9966 | 3.3087 |

Table S4. Adsorption kinetic parameters of astaxanthin on d-UiO-67-4.

|  | Pseudo-first-order kinetic model | | |  | Pseudo-second-order kinetic model | | |  |
| --- | --- | --- | --- | --- | --- | --- | --- | --- |
| C_0_ (mg L^-1^) | *k*_1_ (min^-1^) | *q*_e_ (mg g^-1^), cal | R^2^ |  | *k*_2_ (g mg^-1^ min^-1^) | *q*_e_ (mg g^-1^), cal | R^2^ | *q*_e_, exp |
| 2 | 0.0046 | 0.9908 | 0.9750 |  | 8.6655 | 1.0000 | 0.9913 | 0.9600 |
| 4 | 0.0051 | 1.7960 | 0.9813 |  | 5.1760 | 2.0000 | 0.9995 | 1.9169 |
| 8 | 0.0055 | 3.3212 | 0.9929 |  | 3.6145 | 3.3333 | 0.9973 | 3.4174 |

Table S5. Adsorption kinetic parameters of astaxanthin on d-UiO-67-6.

|  | Pseudo-first-order kinetic model | | |  | Pseudo-second-order kinetic model | | |  |
| --- | --- | --- | --- | --- | --- | --- | --- | --- |
| C_0_ (mg L^-1^) | *k*_1_ (min^-1^) | *q*_e_ (mg g^-1^), cal | R^2^ |  | *k*_2_ (g mg^-1^ min^-1^) | *q*_e_ (mg g^-1^), cal | R^2^ | *q*_e_, exp |
| 2 | 0.0044 | 0.9532 | 0.9320 |  | 8.1103 | 1.0000 | 0.9920 | 0.9039 |
| 4 | 0.0048 | 1.7195 | 0.944 |  | 8.2949 | 1.6667 | 0.9934 | 1.7574 |
| 8 | 0.0051 | 3.1246 | 0.9494 |  | 3.1142 | 3.3333 | 0.9971 | 3.1564 |

Table S6. Adsorption kinetic parameters of astaxanthin on d-UiO-67-8.

|  | Pseudo-first-order kinetic model | | |  | Pseudo-second-order kinetic model | | |  |
| --- | --- | --- | --- | --- | --- | --- | --- | --- |
| C_0_ (mg L^-1^) | *k*_1_ (min^-1^) | *q*_e_ (mg g^-1^), cal | R^2^ |  | *k*_2_ (g mg^-1^ min^-1^) | *q*_e_ (mg g^-1^), cal | R^2^ | *q*_e_, exp |
| 2 | 0.0044 | 0.9460 | 0.9270 |  | 9.4150 | 0.7692 | 0.9958 | 0.8556 |
| 4 | 0.0046 | 1.9249 | 0.8954 |  | 6.1017 | 1.6667 | 0.9966 | 1.5925 |
| 8 | 0.0048 | 3.0339 | 0.9128 |  | 0.7745 | 3.3333 | 0.9927 | 2.3866 |

Table S7. Fitting parameters of internal diffusion model of UiO-67.

| C_0_ | kd_1_ | | C_1_ | R^2^ | kd_2_ | C_2_ | R^2^ |
| --- | --- | --- | --- | --- | --- | --- | --- |
| mg/L | mg/g·min^1/2^ | | mg/g |  |  |  |  |
| 2 | 0.0236 | 0.0210 | | 0.9569 | 0.0095 | 0.2369 | 0.9144 |
| 4 | 0.0393 | -0.0125 | | 0.9941 | 0.0174 | 0.3174 | 0.9663 |
| 8 | 0.0886 | -0.0361 | | 0.9725 | 0.0323 | 0.7181 | 0.9868 |

Table S8. Fitting parameters of internal diffusion model of d-UiO-67-2.

| C_0_ | kd_1_ | | C_1_ | R^2^ | kd_2_ | C_2_ | R^2^ |
| --- | --- | --- | --- | --- | --- | --- | --- |
| mg/L | mg/g·min^1/2^ | | mg/g |  |  |  |  |
| 2 | 0.0394 | 0.00712 | | 0.9441 | 0.0168 | 0.3227 | 0.8424 |
| 4 | 0.0794 | -0.0282 | | 0.9908 | 0.0336 | 0.5970 | 0.9776 |
| 8 | 0.1616 | -0.0542 | | 0.9766 | 0.0464 | 1.6198 | 0.9572 |

Table S9. Fitting parameters of internal diffusion model of d-UiO-67-4.

| C_0_ | kd_1_ | | C_1_ | R^2^ | kd_2_ | C_2_ | R^2^ |
| --- | --- | --- | --- | --- | --- | --- | --- |
| mg/L | mg/g·min^1/2^ | | mg/g |  |  |  |  |
| 2 | 0.0368 | 0.0160 | | 0.96454 | 0.0183 | 0.3093 | 0.8927 |
| 4 | 0.1073 | -0.0763 | | 0.9849 | 0.0236 | 1.0718 | 0.9238 |
| 8 | 0.1649 | 0.0163 | | 0.9879 | 0.0491 | 1.7244 | 0.8204 |

Table S10.Characteristic parameters of internal diffusion model of d-UiO-67-6.

| C_0_ | kd_1_ | | C_1_ | R^2^ | kd_2_ | C_2_ | R^2^ |
| --- | --- | --- | --- | --- | --- | --- | --- |
| mg/L | mg/g·min^1/2^ | | mg/g |  |  |  |  |
| 2 | 0.0369 | 0.0094 | | 0.9290 | 0.0170 | 0.2628 | 0.9790 |
| 4 | 0.0792 | 0.0934 | | 0.9659 | 0.0268 | 0.7562 | 0.9871 |
| 8 | 0.1662 | -0.0331 | | 0.9508 | 0.0430 | 1.5816 | 0.9292 |

Table S11.Characteristic parameters of internal diffusion model of d-UiO-67-8.

| C_0_ | kd_1_ | | C_1_ | R^2^ | kd_2_ | C_2_ | R^2^ |
| --- | --- | --- | --- | --- | --- | --- | --- |
| mg/L | mg/g·min^1/2^ | | mg/g |  |  |  |  |
| 2 | 0.0301 | 0.0223 | | 0.9895 | 0.0178 | 0.1840 | 0.9957 |
| 4 | 0.0542 | 0.0280 | | 0.9923 | 0.0341 | 0.2661 | 0.9767 |
| 8 | 0.0712 | 0.0346 | | 0.8947 | 0.0552 | 0.3297 | 0.9792 |

Table S12.Freundlich Parameters of defective UiO-67 and d-UiO-67-X for AXT Adsorption.

|  | Freundlich | | | |
| --- | --- | --- | --- | --- |
|  | Equation | R^2^ | K_F_ | 1/n |
| UiO-67 | Q_e_ = 0.3929C_e_0.7393 | 0.9770 | 0.3929 | 0.7393 |
| d-UiO-67-2 | Q_e_ = 0.5244C_e_0.9305 | 0.9952 | 0.5244 | 0.9305 |
| d-UiO-67-4 | Q_e_ = 0.5082C_e_0.9682 | 0.9969 | 0.5082 | 0.9682 |
| d-UiO-67-6 | Q_e_ = 0.5196C_e_0.9191 | 0.9921 | 0.5196 | 0.9191 |
| d-UiO-67-8 | Q_e_ = 0.4675C_e_0.9422 | 0.9916 | 0.4675 | 0.9422 |

Table S13.Langmuir Parameters of defective UiO-67 and d-UiO-67-X for AXT Adsorption.

|  | Langmuir | | | |
| --- | --- | --- | --- | --- |
|  | Equation | R^2^ | K_L_ | Q_m_ (mg/g) |
| UiO-67 | C_e_/Q_e_ = 0.0779C_e_+3.4429 | 0.9335 | 0.0226 | 12.8452 |
| d-UiO-67-2 | C_e_/Q_e_ = 0.0100C_e_+2.0522 | 0.8000 | 0.0049 | 99.7009 |
| d-UiO-67-4 | C_e_/Q_e_ = 0.0043C_e_+2.0358 | 0.4051 | 0.0021 | 231.4815 |
| d-UiO-67-6 | C_e_/Q_e_ = 0.0118C_e_+2.1083 | 0.7243 | 0.0056 | 84.6740 |
| d-UiO-67-8 | C_e_/Q_e_ = 0.0099C_e_+2.2501 | 0.5453 | 0.0044 | 100.9082 |

Table S14.Comparison of BET surface area, maximum adsorption capacity and purity of AXT by different adsorbents.

| adsorbent | BET Surface area  (m^2^ g^-1^) | Maximum Adsorption  capacity (mg g^-1^) | Purity (%) | Reference |
| --- | --- | --- | --- | --- |
| XDA-8 | ≥400 | ~2.4 | 14.64% (1^st^)  87.34% (2^rd^) | ^[15]^ |
| LX-68G | ≥950 | ~2.7 | - | ^[15]^ |
| LX-68M | ≥1000 | ~2.05 | - | ^[15]^ |
| LX-69B | ≥980 | ~2.1 | - | ^[15]^ |
| AB-8 | ≥400 | ~2 | - | ^[15]^ |
| D301 | ≥400 | ~1.6 | - | ^[15]^ |
| D101 | ≥520 | ~2.5 | - | ^[15]^ |
| UiO-67 | ≥500 | 7.22 | - | In this work |
| d-UiO-67-4 | ≥1360 | 26.21 | 89.0% ± 2.3% (1^st^) | In this work |

References

[1] X. Zhang, X. Shi, Q. Zhao, Y. Li, J. Wang, Y. Yang, F. Bi, J. Xu, N. Liu, *Chem. Eng. J.* **2022**,*427*, 131573.

[2] Q. Su, W. Su, S. Xing, M. Tan, *Carbohydr. Polym.* **2024**,*326*, 121645.

[3] Q. Liu, J. Ye, Y. Han, P. Wang, Z. Fei, X. Chen, Z. Zhang, J. Tang, M. Cui, X. Qiao, *J. Mol. Liq.* **2021**,*321*, 114477.

[4] Q. Zhao, Z. Zhao, R. Rao, Y. Yang, S. Ling, F. Bi, X. Shi, J. Xu, G. Lu, X. Zhang, *J. Colloid Interface Sci.* **2022**,*627*, 385.

[5] Q. Zhao, X. Chen, G.-L. Zhang, H. Hao, B.-w. Zhu, H.-M. Hou, J. Bi, *ACS Appl. Mater. Interfaces* **2022**,*14*, 29131.

[6] J.-P. Yuan, F. Chen, *Food Chem.* **2000**,*68*, 443.

[7] J.-P. Yuan, F. Chen, *J. Agric. Food Chem.* **1999**,*47*, 31.

[8] a) R. Liu, S. Fei, X. Zhang, Z. Hua, M. Tan, *Chem. Eng. J.* **2024**,*479*, 147590; b) Y. Song, L. Song, F. Yin, J. Zhang, J. Zhang, D. Zhou, T. Wang, *J. Am. Oil Chem. Soc.* **2018**,*95*, 1171.

[9] X. Ren, C.-C. Wang, Y. Li, P. Wang, S. Gao, *J. Hazard. Mater.* **2023**,*445*, 130552.

[10] M. J. Frisch, G. W. Trucks, H. B. Schlegel, G. E. Scuseria, M. A. Robb, J. R. Cheeseman, G. Scalmani, V. Barone, G. A. Petersson, H. Nakatsuji, X. Li, M. Caricato, A. V. Marenich, J. Bloino, B. G. Janesko, R. Gomperts, B. Mennucci, H. P. Hratchian, J. V. Ortiz, A. F. Izmaylov, J. L. Sonnenberg, D. Williams-Young, F. Ding, F. Lipparini, F. Egidi, J. Goings, B. Peng, A. Petrone, T. Henderson, D. Ranasinghe, V. G. Zakrzewski, J. Gao, N. Rega, G. Zheng, W. Liang, M. Hada, M. Ehara, K. Toyota, R. Fukuda, J. Hasegawa, M. Ishida, T. Nakajima, Y. Honda, O. Kitao, H. Nakai, T. Vreven, K. Throssell, J. A., Jr. Montgomery, J. E. Peralta, F. Ogliaro, M. J. Bearpark, J. J. Heyd, E. N. Brothers, K. N. Kudin, V. N. Staroverov, T. A. Keith, R. Kobayashi, J. Normand, K. Raghavachari, A. P. Rendell, J. C. Burant, S. S. Iyengar, J. Tomasi, M. Cossi, J. M. Millam, M. Klene, C. Adamo, R. Cammi, J. W. Ochterski, R. L. Martin, K. Morokuma, O. Farkas, J. B. Foresman, D. J. Fox, Gaussian 16, Revision A.03; Gaussian, Inc., Wallingford CT, **2016**.

[11] a) A. H. Vahabi, F. Norouzi, E. Sheibani, M. Rahimi-Nasrabadi, *Coord. Chem. Rev.* **2021**,*445*, 214050; b) S. Grimme, J. Antony, S. Ehrlich, H. Krieg, *The Journal of chemical physics* **2010**,*132*, 15.

[12] P. Brandt, S.-H. Xing, J. Liang, G. l. Kurt, A. Nuhnen, O. Weingart, C. Janiak, *ACS Appl. Mater. Interfaces* **2021**,*13*, 29137.

[13] G. C. Shearer, S. Chavan, J. Ethiraj, J. G. Vitillo, S. Svelle, U. Olsbye, C. Lamberti, S. Bordiga, K. P. Lillerud, *Chemistry of Materials* **2014**,*26*, 4068.

[14] A. H. Vahabi, F. Norouzi, E. Sheibani, M. Rahimi-Nasrabadi, *Coordination Chemistry Reviews* **2021**,*445*, 214050.

[15] L. Wang, J. Hu, W. Lv, W. Lu, D. Pei, Y. Lv, W. Wang, M. Zhang, R. Ding, M. Lv, *Food Chem.* **2021**,*363*, 130369.
